# The Chitosan-Based System with *Scutellariae baicalensis radix* Extract for the Local Treatment of Vaginal Infections

**DOI:** 10.3390/pharmaceutics14040740

**Published:** 2022-03-29

**Authors:** Justyna Chanaj-Kaczmarek, Natalia Rosiak, Daria Szymanowska, Marcin Rajewski, Ewa Wender-Ozegowska, Judyta Cielecka-Piontek

**Affiliations:** 1 Department of Pharmacognosy, Poznan University of Medical Sciences, 3 Rokietnicka Street, 60-806 Poznan, Poland; justyna.chanaj-kaczmarek@ump.edu.pl (J.C.-K.); nrosiak@ump.edu.pl (N.R.); 2 Department of Biotechnology and Food Microbiology, Poznan University of Life Sciences, 48 Wojska Polskiego Street, 60-627 Poznan, Poland; daria.szymanowska@up.poznan.pl; 3 Department of Reproduction, Poznan University of Medical Sciences, 33 Polna Street, 60-535 Poznan, Poland; rajewskim@wp.pl (M.R.); ewozegow@ump.edu.pl (E.W.-O.)

**Keywords:** *Scutellaria baicalensis*, vaginal infection, dissolution, spectrophotometric method, thermic method

## Abstract

*Scutellarie baicalensis radix*, as a flavone-rich source, exhibits antibacterial, antifungal, antioxidant, and anti-inflammatory activity. It may be used as a therapeutic agent to treat various diseases, including vaginal infections. In this study, six binary mixtures of chitosan with stable *S. baicalensis radix* lyophilized extract were obtained and identified by spectral (ATR-FTIR, XRPD) and thermal (TG and DSC) methods. The changes in dissolution rates of active compounds and the significant increase in the biological properties towards metal chelating activity were observed, as well as the inhibition of hyaluronic acid degradation after mixing plant extract with chitosan. Moreover, the combination of *S. baicalensis radix* lyophilized extract with a carrier allowed us to obtain the binary systems with a higher antifungal activity than the pure extract, which may be effective in developing new strategies in the vaginal infections treatment, particularly vulvovaginal candidiasis.

## 1. Introduction

Bacterial vaginosis (BV) and vulvovaginal candidiasis (VVC) are the most vaginal tract infections diagnosed in reproductive age women, and are caused by a physiological imbalance in the vaginal microflora [1]. BV is characterized by an increase in the vaginal pH due to a reduced number of *Lactobacillus* and an overgrowth of anaerobic bacteria, such as *Gardnerella vaginalis*, *Mycoplasma hominis*, *Prevotella*, and *Peptostreptococcus* [2,3,4]. Furthermore, the small amount of *Lactobacillus* may lead to *Candida* species-induced VVC due to enhancing the growth and the adherence to the vaginal epithelium of *Candida albicans*, *Candida tropicalis*, or *Candida glabrata* [5].

Untreated vaginal infections may be associated with numerous complications, including increasing the risk of sexually transmitted diseases, such as human immunodeficiency virus (HIV), herpes simplex virus (HSV-2), *Chlamydia trachomatis*, and gonorrhea, as well as complications and infections that may threaten pregnancy (late miscarriages, preterm birth, intrauterine infection, endometritis) [6,7,8]. Moreover, BV increases the risk of postoperative gynecological infections following cesarean delivery, hysterectomies, and surgical abortions [9].

Bacterial and fungal infections caused by drug-resistant strains constitute a significant challenge to the effective therapy [10]. Despite the recommended treatment, more than 50% and up to 9% of women experienced a recurrence of BV and VVC, respectively, within the subsequent 12 months [11,12]. Several reports indicated that the biofilm might be responsible for the treatment failure and vaginal infections recurrence [13,14]. Biofilm formation is a dynamic and multi-step process involving multiple interactions between one or several microbial species [15]. Biofilms are multicellular communities of microorganisms held together by self-biosynthesized extracellular polymeric substances (EPS) composed of polysaccharides, proteins, rare sugars, lipids, and deoxyribonucleic acids (DNA) [16,17,18]. Furthermore, the production of EPS plays a fundamental role in the stability of the cells, assists adhesion of the cells to the surface, and protects pathogens from altered pH, osmolarity, or nutrient scarcity [19,20]. Patterson et al. noticed an increased tolerance of *G. vaginalis* biofilms to 5-fold higher hydrogen peroxide concentrations and 4–8-fold higher lactic acid concentrations than planktonic cells using an in-vitro model [21]. The presence of EPS also contributes to blocking the access of antibiotics to pathogen cells leading to slow or incomplete penetration of the antibiotics into the biofilm. This, in turn, results in the failure of therapeutic strategies and gives rise to drug resistance in bacteria [20]. The recurrence cases of vaginal infection require longer-duration treatment, which may extend even to six months and lead to the depletion of the vaginal bacterial microflora [6,22].

Natural products are traditionally used in the treatment of vaginal infection due to their multifunctional therapeutic effects [23,24,25]. In fact, several clinical trials demonstrated that using plant extracts promoted the reduction of vaginal infection symptoms [26,27]. *Scutellaria baicalensis radix* is a plant material with well-documented antibacterial and antifungal properties, including activity against the most common causes of vaginal infection, such as *G. vaginalis* and *C. albicans* [28,29]. The major bioactive compounds from the root of *S. baicalensis*, particularly baicalin, baicalein and wogonin, account for the abovementioned significant pharmacological effects [30]. Additionally, baicalin effectively inhibited *Chlamydia trachomatis* by reducing the expression of protease-like activity factor (CPAF) genes in Hep-2 cells infected by this Gram-negative bacteria [31]. Moreover, baicalin, baicalein, and wogonin may reduce local inflammation associated with the vaginal infection by suppressing the production of inflammatory cytokines through the inhibition of NF-κB activation and inducible nitric oxide synthase [32,33,34,35,36,37].

The biological activity of *S. baicalensis radix* is believed to be also associated with the iron-chelating properties of flavones, which was observed mainly with regard to baicalein. Mladěnka et al. demonstrated that the most effective iron-binding site of baicalein results from C6, C7–dihydroxyl groups and a C2, C3–double bond in the structure of this compound [38]. Iron-chelating capacity plays an important role in the antioxidant activity of *S. baicalensis radix* by reducing the generation of hydroxyl radicals in the Fenton reaction [39]. Interestingly, the hydroxyl radical possesses strong oxidizing properties and is a significant cause of tissue damage and a chronic inflammation in humans [40]. Moreover, several reports have indicated that iron plays a crucial role in the process of microcolony formation and biofilm maturation in some pathogens, such as *Campylobacter jejuni* [41], *Klebsiella pneumoniae* [42], *Pseudomonas aeruginosa* [43], and *Staphylococcus aureus* [44]. Thus, iron is a vital nutrient for bacterial growth and is necessary for energy production, nucleotide synthesis, and regulation of gene expression by pathogens [44]. In addition, to avoid an extreme iron deficiency, bacteria produce iron chelators, referred to as siderophores, in order to scavenge iron from the extracellular environment [45].

Hyaluronic acid is composed of alternating units of D-glucuronic acid and N-acetyl-D-glucosamine [46]. It is found in various body tissues and fluids, such as the eyes, joints, synovial fluids, and extracellular matrix [47], and it plays a crucial role in the inflammatory response, angiogenesis, and tissue regeneration [48]. It protects tissues against oxidative damage by neutralizing reactive oxygen species, such as superoxide anion, hydrogen peroxide, and hydroxyl ion [49]. Moreover, hyaluronic acid may dose-dependently inhibit bacterial growth, reduce pathogens adhesion and biofilm formation [50]. Therefore, due to hyaluronic acid properties, including modulating tissue hydration and acting as a lubricant, it is widely used to develop topical applications used for non-hormonal treatments for the signs of post-menopausal vaginal atrophy, dyspareunia, and the reduction of side effects due to cervical cancer radiotherapy [51,52,53].

Chitosan is a natural polymer obtained by alkaline deacetylation of chitin [54]. It has demonstrated excellent biological properties, such as antibacterial, antifungal, and wound-healing activity [55,56,57,58]. The highest antimicrobial effects of chitosan as a cationic polymer are observed in an acidic environment [59]. In view of the mucoadhesive properties of chitosan, it is widely used to develop vaginal controlled release drug delivery systems applied topically [60]. The mucoadhesive properties of the polymer stem from the ionic interactions of its protonated amino groups with the negatively charged sialic acid groups on mucin [61]. Moreover, chitosan presents penetration enhancement properties for poorly soluble compounds by the mucosal membrane [54], which is attributed to an increased dissolution through amorphization and a decreased aggregation of the compounds [62], as well as improving transport by transiently and reversibly opening the tight junctions integrity of the biological membranes [63].

The presented study aimed to evaluate the effectiveness of the chitosan-based system with *S. baicalensis radix* lyophilized extract towards inhibition hyaluronic acid degradation, metal chelating activity, and the effects on the vaginal microbiome. For the characterization of the obtained chitosan-based binary systems, the spectroscopic (XRPD and ATR-FTIR) and thermal (TG and DSC) methods were used.

## 2. Materials and Methods

### 2.1. Chemicals and Reagents

Baicalin, baicalein, and wogonin as phyproof^®^ reference substances were purchased from Sigma-Aldrich Co. (St Louis, MO, USA). The following chemicals from Sigma-Aldrich were used: sodium chloride, potassium hydroxide, calcium hydroxide, lactic acid, bovine serum albumin, acetic acid, glycerol, urea, glycerol, iron(II) chloride, ferrozine iron reagent, hyaluronidase, and hyaluronic acid. Chitosan (Cs) 80/500 (degree of deacetylation: 77.6–82.5%; viscosity: 351–750 mPas) and 80/1000 (degree of deacetylation: 77.6–82.5%; viscosity: 751–1250 mPas) were supplied by Heppe Medical Chitosan GmbH (Halle, Germany). HPLC grade methanol, acetic acid, and acetate buffer were provided by the JT Baker–Avantor Performance Materials B.V. (Deventer, The Netherlands). High-quality pure water and ultra-high-quality pure water were prepared using the Direct-Q 3 UV Merck Millipore purification system (Merck, Darmstadt, Germany).

### 2.2. Flavones Content in S. baicalensis radix Lyophilized Extract

The standardized lyophilized extract of *S. baicalensis radix* (SBE) was used for the study regarding the content of the three flavones that play a crucial role in the biological activity of the plant material. Using the high-performance liquid chromatography with diode array detector (HPLC-DAD) method, the determined content of baicalin was 178.10 ± 1.90 mg per 1 g of lyophilized extract, and the content of baicalein was 62.93 ± 1.23 mg per 1 g of lyophilized extract, whereas that of wogonin was 25.31 ± 0.19 mg per 1 g of the lyophilized extract [64].

### 2.3. Stability Testing of S. baicalensis radix Lyophilized Extract

The stability of the lyophilized extract of *S. baicalensis* root was estimated according to the International Conference on Harmonization (ICH Q1A(R2)) guidelines regarding the stability testing of new drug substances and products stored at room temperature [65]. The analysis of stability samples was conducted for the intermediate conditions [30 °C ± 2 °C, 65% relative humidity ± 5%] for six months. The quantification of flavones from samples taken every month was determined using the HPLC-DAD method.

### 2.4. Preparation of the Binary Systems

The 60% hydroalcoholic solutions of *S. baicalensis radix* lyophilized extract were added to 1% acetic acid solutions of chitosan (Cs) in a weight ratio SBE:Cs of 2:1, 1:1, and 1:2; subsequently, the mixtures were stirred for 4 h by means of a magnetic stirrer at 1000 rpm. The study used two types of chitosan with a degree of deacetylation of 80% and different viscosity (500 mPas and 1000 mPas). The hydroalcoholic solution of *S. baicalensis radix* lyophilized radix was prepared using an ultrasonic bath Elma S180H (Elma Ultrasonic Technology, Singen, Germany) at a temperature of 40 °C for 30 min, whereas the 1% acetic acid solution of chitosan was stirred overnight at 150 rpm (Sunlab SU1300) at room temperature to solubilize the chitosan completely. Next, the obtained mixtures of *S. baicalensis radix* extract and chitosan were evaporated using BÜCHI Rotavapor R-210 at 35 °C to a syrupy consistency, were frozen, and then were lyophilized. The freeze-drying was conducted at reduced pressure (2–9 hPa). The condensation temperature was set to −55 °C for 48 h (Heto PowerDry PL3000, Thermo Fisher Scientific, Waltham, MA, USA). The six different binary mixtures in the form of powders were stored at room temperature.

### 2.5. Identity Study of the Binary Systems

#### 2.5.1. Fourier-Transform Infrared Spectroscopy (ATR-FTIR) and Density Functional Theory (DFT) Calculations

The ATR-FTIR spectra were collected on an IRTracer-100 spectrophotometer. All spectra were measured between 4000 and 400 cm^−1^ in the absorbance mode. The following spectrometer parameters were used: resolution: 4 cm^−1^, the number of scans: 400, and apodization: Happ–Genzel. The sample was placed directly on the ATR crystal. Solid samples were pressed against the ATR crystal and the ATR-FTIR spectrum was scanned. All IR spectra were acquired and further processed with LabSolution IR software. The results were interpreted by comparing the FTIR peaks of pure samples with those of the prepared binary systems. Origin 2021b (OriginLab Corporation, Northampton, MA, USA) was used to analyze the acquired data. LabSolutions IR software (version 1.86 SP2, Shimadzu, Kyoto, Japan) was used to calculate the second derivative of spectra for *S. baicalensis radix* extract, baicalein, baicalin, and wogonin. The software performed calculations according to the Savitzky–Golay numerical algorithm. The smoothing parameter was 25 points. A derivative spectrum allowed us to identify the peak positions of the original spectrum and to separate multiple peaks, which were adjoining or on the shoulder. The minima of the second derivative corresponded to the extremes of the original ATR-FTIR spectrum.

The molecular geometries were optimized using the Density Functional Theory (DFT) method with Becke’s three-parameter hybrid functional (B3LYP), implemented with the standard 6-311G(d,p) as a basis set. Additionally, the calculations of the normal mode frequencies and intensities were also performed. The PL-Grid platform (website: www.plgrid.pl, accessed on 1 December 2021) with the Gaussian 09 package (Wallingford, CT, USA) [66] was used for all DFT calculations. The GaussView (Wallingford, CT USA, Version E01) [67] program was used to propose an initial geometry of the investigated molecules and for the visual inspection of the normal modes. The obtained data were analyzed by means of the Origin 2021b software.

#### 2.5.2. X-ray Powder Diffraction (XRPD)

Diffraction patterns were recorded on a PANalitycal Empyrean diffractometer (Malvern Panalytical, Malvern, UK) with CuKα radiation (1.54056 Å) at a tube voltage of 45 kV and a tube current of 40 mA. The angular range was 3° to 50°, with a step size of 0.017° and a counting rate of 15 s step^−1^. Origin 2021b was used to analyze the acquired data.

#### 2.5.3. Thermogravimetric Analysis (TG)

Thermogravimetric (TG) analysis and differential (DTG) thermogravimetric analysis were performed using TG 209 F3 Tarsus^®^ micro-thermobalance (Netzsch, Selb, Germany). Subsequently, about 6 mg powdered samples were placed in Al_2_O_3_ 85 µL, open and heated at a scanning rate of 10 °C min^−1^ from 25 to 900 °C in a nitrogen atmosphere with a flow rate of 250 mL min^−1^. The obtained TG data were analyzed using the Proteus 8.0 (Netzsch) computer program.

#### 2.5.4. Differential Scanning Calorimetry (DSC)

Thermal analysis was performed using the DSC 214 Polyma differential scanning calorimeter (Netzsch, Selb, Germany). Next, about 6 mg powdered samples were placed in the crimped aluminum pans with a small hole in the lid, and were heated at a scanning rate of 10 °C min^−1^ from 25 to 280 °C in a nitrogen atmosphere with a flow rate of 30 mL min^−1^. The obtained DSC data were analyzed (determination of enthalpy, phase transition temperatures) using the Proteus 8.0 (Netzsch) computer program.

### 2.6. Dissolution Studies

The dissolution profiles of flavones from the *S. baicalensis radix* extract and the binary systems were determined in 150 mL of the vaginal fluid simulant solution, prepared according to Owen et al. [68] and composed (per liter) of sodium chloride (3.51 g), potassium hydroxide (1.40 g), calcium hydroxide (0.222 g), bovine serum albumin (0.018 g), lactic acid (2.00 g), acetic acid (1.00 g), glycerol (0.16 g), urea (0.4 g), and glucose (5.0 g). The samples were tested using a standard paddle Agilent 708-DS dissolution apparatus with a stirring speed of 50 rpm and at a temperature of 37 ± 0.5 °C [69]. Sink conditions were maintained throughout the tests. The dissolution samples (2.0 mL) were collected at the appropriate time intervals for 24 h, and replaced by equal volumes of the pre-warmed acceptor solution. Each sample was immediately filtered through a 0.45 μm membrane filter. The concentration of active compounds in the vaginal fluid simulant solution was determined by the HPLC-DAD method [64]. The similarity of dissolution percentage of the active compounds from the binary mixtures was established based on *f*_1_ and *f*_2_ parameters and was defined by the following equation:

(1) $$f_{1}=\frac{\sum_{j=1}^{n}\left|R_{j}-T_{j}\right|}{\sum_{j=1}^{n}R_{j}}\times 100$$

(2) $$f_{2}=50\times \log\left({\left(1+\left(\frac{1}{n}\right)\sum_{j=1}^{n}{\left|R_{j}-T_{j}\right|}^{2}\right)}^{-\frac{1}{2}}\times 100\right)$$

in which *n* is the number of withdrawal points, *R_j_* is the percentage dissolved of a reference product at time point *t*, and *T_j_* is the percentage dissolved of test product at time point *t*. The *f*_1_ value close to 0 and the *f*_2_ value close to 100 indicate profile similarity [70].

### 2.7. Biological Activity

The solutions to determine the activity towards ferrous ion chelating activity and inhibition of hyaluronidase were prepared by shaking (400 rpm min^−1^) *S. baicalensis radix* lyophilized extract and the binary systems with an acetate buffer solution at pH 4.5 for 45 min at 37 °C on a shaker (ThermoScientific MaxQ 4450, MA, USA). Subsequently, the suspensions were centrifuged at 3500 rpm min^−1^ for 30 min (Nüve NF 800, Ankara, Turkey) to produce a clear supernatant. The IC_50_ values were calculated with OriginPro 9 software (OriginLab Corporation, Northampton, MA, USA). All experiments were performed six times.

#### 2.7.1. Ferrous Ion-Chelating Activity

The chelating ability on ferrous ions of the *S. baicalensis radix* extract and the binary systems was estimated by the method of Dinis with certain modifications [71]. In short, 10 μL of 1 mM FeCl_2_ was mixed with 0.2 mL of the sample at different concentrations. The reaction was initiated by the addition of 10 μL of 2.5 mM ferrozine solution. The final concentration for *S. baicalensis radix* and the binary systems in the samples were 0.91–11.82 mg mL^−1^ and 0.82–7.36 mg mL^−1^, respectively. The chitosans were examined at the concentration of 1–10 mg mL^−1^. The control blank contained acetate buffer at pH 4.5 instead of the investigated solution. The absorbance of the mixture was measured using a spectrophotometer Thermo Scientific Multiskan GO (Thermo Fisher Scientific, Waltham, MA) at 562 nm after incubating for 30 min at room temperature. The metal chelating activity was calculated as a percentage of the inhibition of ferrozine–Fe^2+^ complex formation, according to the following formula:

Metal chelating activity (%) = (Acontrol − Asample)/Acontrol × 100(3)

where Acontrol is the absorbance of the control, and Asample is the absorbance of the tested sample.

#### 2.7.2. Anti-Hyaluronidase Activity

The inhibitory activity on hyaluronidase was evaluated according to the turbidimetric method described by Studzińska-Sroka et al. [72]. The final concentrations for the lyophilized extract, binary systems, and chitosans in samples were in the range of 1.4–2.4 mg mL^−1^, 0.05–0.26 mg mL^−1^, and 0.02–0.06 mg mL^−1^, respectively.

#### 2.7.3. Effects on the Vaginal Microflora

All microorganism strains were inoculated in Müeller-Hinton broth (pH 7.4.) for approximately 16 h. The concentration of the suspensions was adjusted to 0.5 (optical density) by means of a spectrophotometer. Antimicrobial activity of the *S. baicalensis radix* lyophilized extract and the binary systems were determined by the Agar well diffusion method against pathogenic bacteria (*Gardnerella vaginalis* ATCC 14018, *Streptococcus agalactiae* ATCC BAA-611, *Staphylococcus aureus* ATCC 25923, *Escherichia coli* ATCC 25922), probiotic bacteria (*Lactobacillus gasseri* ATCC 33323, *Lactobacillus jensenii* ATCC 25258, *Lactobacillus plantarum* ATCC 814), and yeast-like fungi (*Candida albicans* ATCC 3153, *Candida parapsilosis* ATCC 2195, *Candida krusei* ATCC 573). The 20 mL of sterilized Nutrient Agar was poured into sterile petri plates. Following solidification, 100 μL of standardized inoculate of each isolate was inoculated on Nutrient agar plates using sterilized spreaders. The wells were punched over the agar plates using a sterile gel puncher of 6 mm diameter. A measure of 100 μL of the lyophilized extract and binary systems was poured into separate wells. *S. baicalensis radix* extract and the binary systems were dissolved in 1% (*v*/*v*) dimethylsulphoxide (DMSO), which was used as a negative control. Binary systems were tested at the concentration of 1200 μg mL^−1^, whereas *S. baicalensis radix* lyophilized extract and chitosan 80:500 and 80:1000 were tested at 400, 600, and 800 μg mL^−1^. Metronidazole and clindamycin were used as reference standards at the concentration of 1000 μg mL^−1^. Plates were incubated at 37 °C for 24 h. Triplets of the experiment were maintained for each bacterial strain to ensure reliability. Following incubation, the diameter of the circular inhibitory zones formed around each well was measured in mm and recorded.

### 2.8. Statistical Analysis

The obtained results were analyzed using a one-way analysis of variance (ANOVA) and the statistical significance was determined using Duncan’s post hoc test (*p*-value < 0.05). All statistical analyses were performed using STATISTICA v. 13 (StatSoft, Inc. 2015, Kraków, Poland). All results are presented as the mean ± standard deviation.

## 3. Results and Discussion

### 3.1. Preparation of Binary Systems of S. baicalensis radix Extract with Chitosan

As the first stage of the experimental work, chitosan-based binary systems were prepared using the formation in solution. The solutions of *S. baicalensis radix* lyophilized extract (SBE) and chitosans (Cs), with the same degree of deacetylation (80%) and different viscosity (500 or 1000 mPas), were mixed in the weight ratio SBE:Cs of 2:1 (SBE/Cs 80:500 2:1 and SBE/Cs 80:1000 2:1), 1:1 (SBE/Cs 80:500 1:1 and SBE/Cs 80:1000 1:1), and 1:2 (SBE/Cs 80:500 1:2 and SBE/Cs 80:1000 1:2). According to the International Conference on Harmonization guidelines [65], the standardized plant extract on flavones content [64] was investigated for stability. Feng et al. reported that the degradation of baicalin and baicalein in phosphate buffer solutions depended on pH and temperature. The acidic environments (pH 2–4.5) and the temperature <4 °C were conducive to stabilizing them. Additionally, the protective effect of the coexistent compounds in *S. baicalensis radix* extract against degradation of flavones at pH 6.8 and pH 7.4 was observed [73]. As shown in Table 1, after exposure of *S. baicalensis radix* lyophilized extract to high temperatures (30 °C) and high humidity (65% RH), a 10% reduction in baicalin, baicalein, and wogonin content was observed following six months.

### 3.2. Identity Study of the Binary Systems

#### 3.2.1. Fourier-Transform Infrared Spectroscopy (ATR-FTIR) and Density Functional Theory (DFT) Calculations

The calculations and the experimental IR absorption spectra of baicalin (Figure S1), baicalein (Figure S2), and wogonin (Figure S3) are presented in the supplementary material. In addition, in order to enhance the clarity of the study, band assignments and complete spectroscopic characteristics for baicalin (Table S1), baicalein (Table S2), and wogonin (Table S3) are included in the supplementary material.

For a more accurate identification of baicalin, baicalein, and wogonin from the *S. baicalensis radix* lyophilized extract, second derivative (SD) infrared spectra by the Savitzky–Golay polynomial fitting method were employed to increase the apparent spectral resolution. In the region of 1000–1800 cm^−1^, more peaks can be observed in the spectra (Figure S4, dashed line) [74,75].

Figure S5 shows the second derivative infrared spectra of the *S. baiacalensis radix* extract and the standards. SD spectra of the extract possess more observable corresponding peaks and present similar absorption characteristics to baicalin, baicalein, and wogonin. The most characteristic peaks are described in the supplementary material.

**Figure 1:**
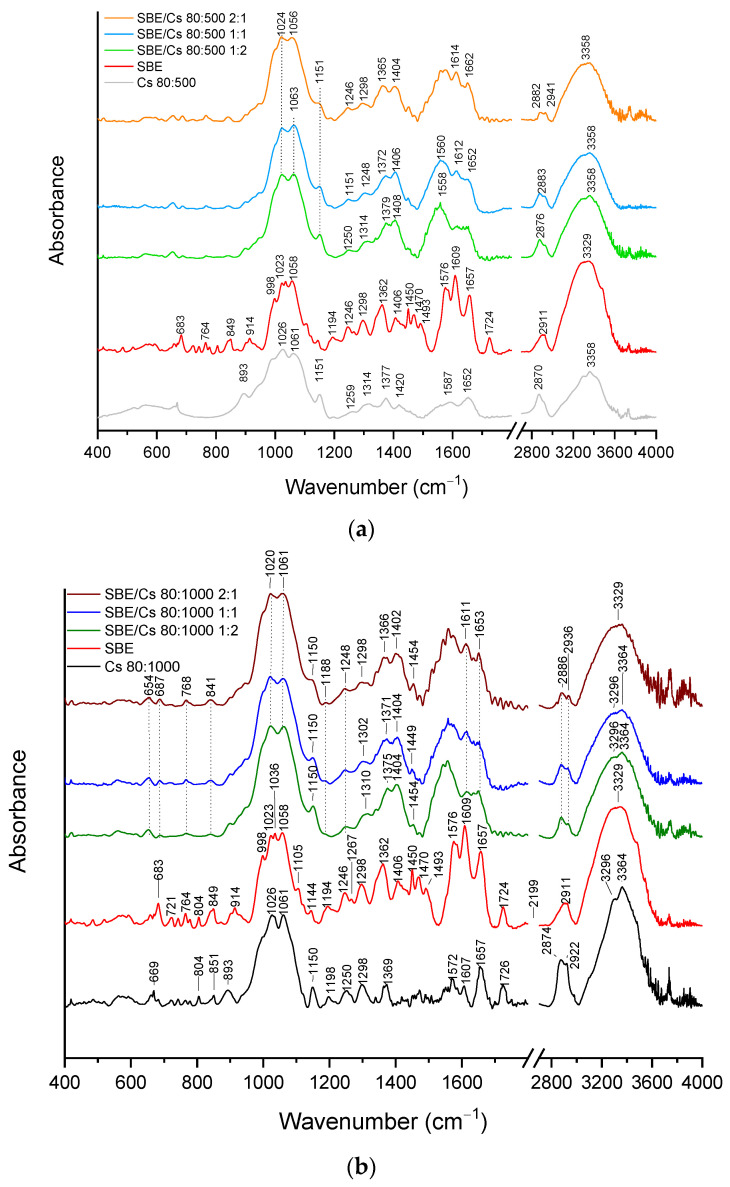
ATR-FTIR spectra of *S. baicalensis radix* extract and the binary systems with (**a**) chitosan 80:500 and (**b**) chitosan 80:1000.

ATR-FTIR spectra of the systems of the *S. baicalensis radix* lyophilized extract with chitosan 80:500 and chitosan 80:1000 are presented in Figure 1a,b, respectively. In terms of chitosan, the 80:500 and the 80:1000 are the most characteristic, whereas IR absorbance peaks are in the range from 897–1650 cm^−1^ and 2800–3450 cm^−1^. The bands corresponding to the stretching vibration of Cs 80:500/Cs 80:1000 are located at 1026/1026 cm^−1^, 1061/1061 cm^−1^ (C–O), 1150/1150 cm^−1^ (C–O–C, asymmetric), 1314/1338 cm^−1^ (C–N, of amide III), 1587/1572 cm^−1^ (amide II peak), 1652/1657 cm^−1^ (C=O, of amide I), 2870/2874 cm^−1^ (C–H, symmetric) -/2922 cm^−1^ (C–H, asymmetric), 3293/3296 cm^−1^ (O–H), and 3358/3364 cm^−1^ (N–H). Next, the bands corresponding to the bending vibration are located at 893/893 cm^−1^ (C–H, out of plane), 1259/1250 cm^−1^ (hydroxyls), 1420/- cm^−1^ (CH_2_), and 1587/1572 cm^−1^ (N–H, of the primary amide) [76].

The literature reports that the most characteristic peaks for the *S. baicalensis radix* lyophilized extract are located at the range of 700–825 cm^−1^ (C–H bending), 1058 cm^−1^ (C–O stretching), 1194 cm^−1^ (C–C skeleton vibration), 1246 cm^−1^ (C–N stretching), 1362 cm^−1^ (vibrations from methyl groups), 1406 cm^−1^ (vibrations from methylene groups), 1450 cm^−1^, 1470 cm^−1^, 1493 cm^−1^, 1576 cm^−1^ (skeleton vibration of benzene), 1609 cm^−1^, and 1657 cm^−1^ (C=O stretching) [77,78]. On the basis of the second derivative infrared spectra and the DFT study, we suggested that baicalin, baicalein, and wogonin bands were assigned to the *S. baicalensis radix* extract bands. Baicalin bands in the spectra of *S. baicalensis radix* extract are found at 683 cm^−1^, 849 cm^−1^, 998 cm^−1^, 1023 cm^−1^, 1058 cm^−1^, 1105 cm^−1^, 1298 cm^−1^, 1406 cm^−1^, 1576 cm^−1^, 1609 cm^−1^, and 1657 cm^−1^. Next, baicalein bands in *S. baicalensis radix* extract are found at 683 cm^−1^, 1023 cm^−1^, 1058 cm^−1^, 1105 cm^−1^, 1298 cm^−1^, 1493 cm^−1^, 1576 cm^−1^, 1609 cm^−1^, and 1657 cm^−1^, whereas wogonin bands are observed at 764 cm^−1^, 849 cm^−1^, 998 cm^−1^, 1058 cm^−1^, 1246 cm^−1^, 1356 cm^−1^, 1493 cm^−1^, 1576 cm^−1^, 1609 cm^−1^, and 1657 cm^−1^ (Tables S4–S6).

The interactions between the *S. baicalensis radix* extract and chitosan were confirmed in ATR-FTIR spectra for the binary systems. The most crucial bands for the extract, chitosan 80:500, and the binary systems with chitosan 80:500 are presented in Table S7. In the spectra of SBE/Cs 80:500, in the range of 660–850 cm^−1^, disappearance of the bands and change in the shape of the bands are observed. For instance, the band observed at 849 cm^−1^ in the *S. baicalensis radix* extract (C–H w (ring A)-baicalin or O–H w (ring A)-wogonin) shows disappearance for the binary systems. In the binary systems with Cs 80:500, shifts and/or disappearance of bands characteristic for the extract are recorded. For example, changes are at 1058 cm^−1^ (C–O–C-baicalin or C–H-baicalein, shift in all systems), 1298 cm^−1^ (C–H and O–H-baicalin or O–H-baicalein, shift in 2:1 system, in the binary systems, in weight ratio 1:1 and 1:2, peak completely disappear), 1362 cm^−1^ (C–H-wogonin, shift in 2:1 system, in 1:1 and 1:2 systems peak completely disappear), 1450 cm^−1^ (O–H, C–O, C–H-baicalin or O–H-baicalein, shift in 2:1 system, in 1:1 and 1:2 systems peak completely disappear), and 1657 cm^−1^ (C=C, C=O-baicalin or C=C, C=O-baicalein or C–C, C=O-wogonin, shift in 2:1 system, in binary systems, in weight ratio 1:1 and 1:2 peak completely disappear). The next bands observed at 914, 998, 1023, 1493, and 1724 cm^−1^ for *S. baicalensis radix* lyophilized extract in all binary systems disappear completely (Table S7). Analogous changes are observed for the binary system of the SBE/Cs 80:1000 systems. In terms of the extract, Cs 80:1000, and SBE/Cs 80:1000 systems, the most important bands are shown in Table S8. The changes observed in the ATR-FTIR spectra may indicate interactions between the extract and chitosan. Furthermore, hydrogen bonds are likely to form between *S. baicalensis radix* lyophilized extract and chitosan.

Less intense and shifting peaks corresponding to the C–O stretching vibrations in chitosan, as well as the changes observed in the intensity of peaks at 3358 cm^−1^ (increases N–H stretching, Cs 80:500) in the binary systems suggest a decrease in particle sizes. In fact, Dennis et al. indicate that C–O stretching vibration was less intense in chitosan nanoparticles and vibrations of N–H increase as the particle sizes decreased [79].

#### 3.2.2. X-ray Powder Diffraction (XRPD)

**Figure 2:**
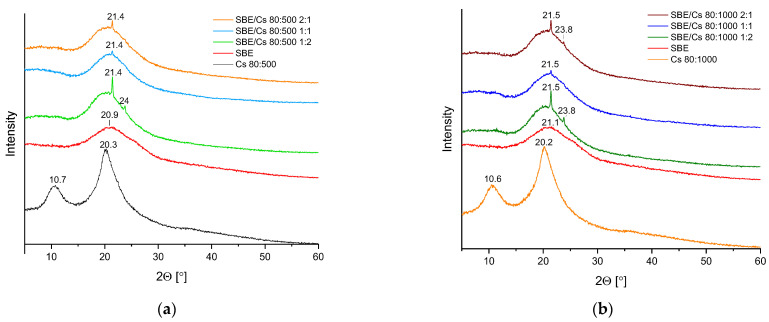
X-ray powder diffraction (XRPD) diffraction patterns of *S. baicalensis radix* extract, chitosan, and the binary systems with (**a**) chitosan 80:500 and (**b**) chitosan 80:1000.

The powder XRPD patterns of the extract, chitosan, and the binary systems are presented in Figure 2a,b. The *S. baicalensis radix* lyophilized extract diffractogram demonstrates one broadened peak around 21° 2θ, whereas XRPD patterns of Cs 80:500 and Cs 80:100 show two peaks around 11° and 20° 2θ. In all diffractogram patterns of the binary systems, the peak shape characteristic of the extract is retained; however, it is worth noting that additional peaks are also present at around 21° 2θ (in all the binary systems) and 24° 2θ (in SBE/Cs 80:500 1:2, SBE/Cs 80:1000 2:1 and 1:2). In fact, they are likely to be nanocrystalline peaks derived from chitosan. Thamilarasan et al. characterized chitosan nanoparticles using XRPD, and observed peaks at around 23 and 26° 2θ [80]. Nevertheless, the method used to prepare the binary system could have influenced the formation of chitosan nanocrystals. According to Zhao et al., lyophilization was indicated as a common technique in the fabrication of chitosan nanoparticles [81]. Additionally, with increasing amounts of chitosan in the binary systems, the increased intensity of these crystalline peaks was also observed.

#### 3.2.3. Thermogravimetric Analysis (TG)

**Figure 3:**
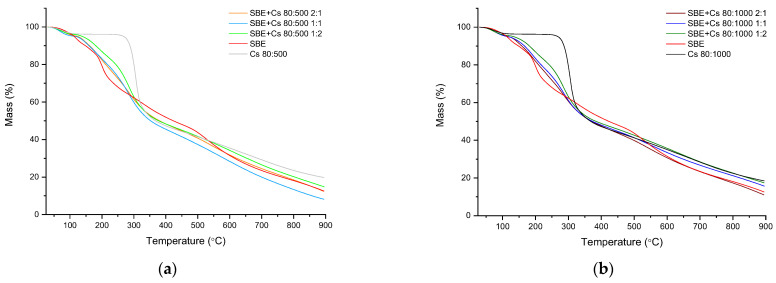
Thermogravimetric analysis at a heating rate of 10 °C min^−1^ of the binary systems (**a**) with chitosan 80:500; (**b**) with chitosan 80:1000.

The TG curves show mass losses in three consecutive steps between 25 °C and 900 °C (Figure 3). In order to improve the readability of the TG curve, the differential thermogravimetric analysis (DTG) was performed in parallel. Figures S6–S14 show the TG curves and the first derivative of the thermogravimetric curve against temperature (T). The inflection points on the DTG curve corresponding to the sample loss are summarized in Table S8. On the basis of the DTG curve, four thermal effects are distinct in *S. baicalensis radix* lyophilized extract curve: two for the Cs 80:500 and Cs 80:1000 curves (first: loss of water; second: corresponds to the decomposition (thermal and oxidative) of chitosan, vaporization, and elimination of volatile products) [82], and three for all binary systems curves. In Figure 3a, the first step observed up to approximately 77 °C (SBE/Cs 80:500 2:1), 70 °C (SBE/Cs 80:500 1:1 and 1:2), 119 °C (SBE), and 78 °C (Cs 80:500) corresponding to loss of water (mass lost = 5%). The second step, up to 285 °C, occurs equally fast for SBE/Cs 80:500 2:1 and SBE/Cs 80:500 1:1. In addition, the thermal decomposition is slower for SBE/Cs 80:500 1:2 due to a greater amount of chitosan in the sample. The third step is in the range of 275–900 °C. From around 305 to 467 °C, the process occurs equally as fast for SBE/Cs 80:500 2:1 and SBE/Cs 80:500 1:2. The residual mass at 900 °C is 12.1%, 8.9%, 14.7%, 19.6%, and 12.3% (SBE/Cs 80:500 2:1, SBE/Cs 80:500 1:1, SBE/Cs 80:500 1:2, Cs 80:500, and SBE, respectively).

In Figure 3b, the first step observed up to approximately 74 °C, 71 °C, 70 °C (SBE/Cs 80:1000 2:1, 1:1, 1:2 respectively), 119 °C (SBE), and 71 °C (Cs 80:1000), corresponding to the water loss (mass lost = 5%). The second step, up to 300 °C, occurs in different speeds for all the binary systems samples. In this range, the thermal decomposition is the slowest for SBE/Cs 80:500 1:2, and the fastest for SBE/Cs 80:500 2:1 (due to the amount of chitosan in the sample). The third step is in the range of 300–900 °C. From around 300 to 403 °C, the process occurs equally as fast for SBE/Cs 80:1000 2:1 and SBE/Cs 80:1000 1:2. The residual mass at 900 °C is 10.8%, 15.5%, 17.2%, 18.3%, and 12.3% (SBE/Cs 80:1000 2:1, SBE/Cs 80:1000 1:1, SBE/Cs 80:1000 1:2, Cs 80:1000, and SBE, respectively).

The differential thermogravimetric analysis shows the first effect, corresponding to the water loss. The second point on the DTG curve for the binary systems can be assigned to *S. baicalensis radix* lyophilization extract; however, we observed that it shifted to a lower temperature (Table S7). The third point can be assigned to chitosan. Similar to the second point, this one is also shifted to lower temperatures in all the binary systems. The last point on the DTG curve, corresponding to pure extract, is observed only in SBE/Cs 80:500 and SBE/Cs 80:1000 in the weight ratio 2:1. In other binary systems, this peak is not clearly observed. The changes observed in the DTG curves (point shift to lower temperatures) may be related to the addition of nanocrystalline chitosan, the presence of which was suggested on the basis of XRPD analysis (Section 3.2.2). In fact, Zhao et al. point out that during the production of nanocrystalline chitosan by the lyophilization method, a sample with minimal thermal stability can be obtained [81].

#### 3.2.4. Differential Scanning Calorimetry (DSC)

**Figure 4:**
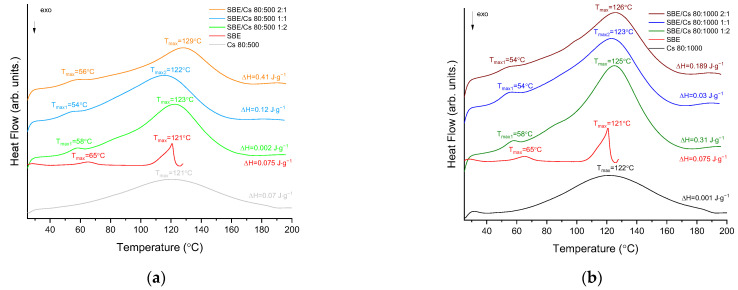
DSC thermograms at a heating rate of 10 °C min^−1^ of the binary systems (**a**) with chitosan 80:500; (**b**) with chitosan 80:1000.

The DSC thermograms derived for *S. baicalensis radix* lyophilized extract, chitosans, and the binary systems are presented in Figure 4. In the DSC thermogram obtained for the extract, two distinct endothermic peaks are visible. The first peak was observed with a maximum at T_1_ = 54 °C, while the second was at T_2_ = 122 °C. The thermal profile of chitosan 80:500 and chitosan 80:1000 exhibited a broad endothermic peak with a maximum at 121 °C and 122 °C, respectively. The peaks are attributed to the water loss associated with the hydrophilic groups of chitosan [76,83].

In the thermal profile of the binary systems with chitosan 80:500 (Figure 4a), two peaks were observed corresponding to the *S. baicalensis radix* extract (first peak at 56 °C, 54 °C, and 58 °C, ratios 2:1, 1:1, and 1:2, respectively) and chitosan (second peak at 129 °C, 122 °C, and 123 °C, ratios 2:1, 1:1, and 1:2, respectively). In the thermal profiles of the binary systems with chitosan 80:1000 (Figure 4b), two peaks were found corresponding to the extract (first peak at 54 °C, 54 °C, and 58 °C, ratios 2:1, 1:1, and 1:2, respectively) and chitosan (second peak at 126 °C, 123 °C, and 125 °C, ratios 2:1, 1:1, and 1:2, respectively). In view of the fact that the ATR-FTIR studies indicate the interaction between the two components (Section 3.2.1), it seems likely that the changes observed in the DSC thermogram (a shift of endothermic effects) correspond to the interaction between chitosan and the *S. baicalensis radix* lyophilized extract in all the studied systems. In fact, Liao et al. indicated that a shift the endothermic peak in chitosan/polycaprolactam blends confirms a formed hydrogen bond between the two components [84].

### 3.3. Dissolution Studies

**Figure 5:**
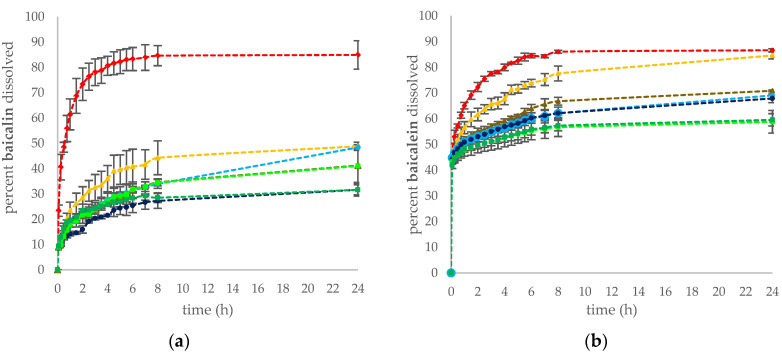

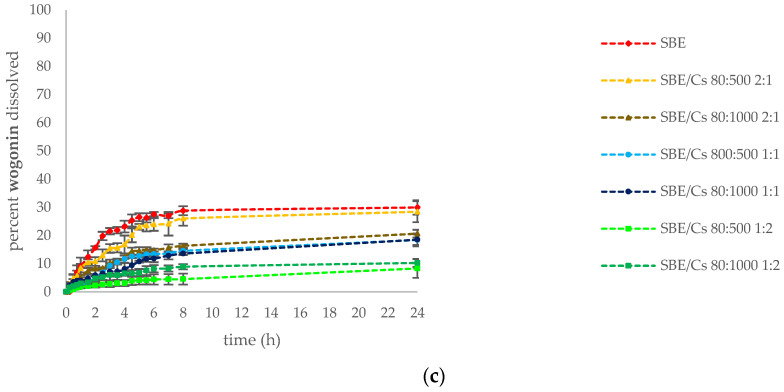
Dissolution profiles of (**a**) baicalin, (**b**) baicalein, and (**c**) wogonin from pure *S. baicalensis radix* lyophilized radix and the binary systems.

Studies have demonstrated the potential of mucoadhesive properties of chitosan in the design of a local application drug delivery system, which prolongs the drug residence time on the surface of the vaginal mucosa [85,86]. The comparative release profiles of flavones in the binary systems for 24 h in the vaginal fluid simulant solution are shown in Figure 5. The release profiles obtained from flavonoids of pure *S. baicalensis radix* lyophilized extract were used as a reference. The dissolution profiles of all formulations indicated that the release of the active compounds decreased with an increase of the quantity of chitosan in the binary systems. The binary systems with chitosan amounts in the weight ratio SBE:Cs of 1:2 showed the lowest baicalin, baicalein, and wogonin release profiles, which at the 24 h of the study were in the range (%) of 31.61 ± 1.99–41.02 ± 6.56, 58.80 ± 4.39–59.56 ± 2.39, and 8.29 ± 3.30–10.24 ± 1.42, respectively. In terms of the binary system, for SBE/Cs 80:500 in the weight ratio of 2:1, the weakest prolonged release of active compounds was observed, in particular for baicalein and wogonin, which was 84.60 ± 1.36 and 28.41 ± 3.74 at 24 h of the study. Moreover, a poorer dissolution of baicalin from the binary systems may result from chitosan swelling at an acidic medium due to the protonation of amino groups. It was demonstrated that the diffusion of the entrapped large molecular weight molecules in the chitosan gels is released mainly due to polymer erosion [87]. Additionally, it is suspected that the initial rapid release of active compounds may stem from the dissolution of flavonoids from the surface of the carrier [88].

### 3.4. Biological Activity

#### 3.4.1. Ferrous Ion-Chelating and Anti-Hyaluronidase Activity

The next step was to evaluate action towards the metal chelating and anti-hyaluronidase activity of the binary systems compared to the pure *S. baicalensis radix* lyophilized extract, which may be effective in the vaginal infection treatment. Several reports indicated the anti-biofilm and antimicrobial properties of a metal chelator, such as EDTA, against the pathogenic bacteria, e.g., *Staphylococcus aureus*, methicillin-resistant *S. aureus* (MRSA), *S. epidermidis*, *Pseudomonas aeruginosa*, and *Enterococcus faecalis* [89,90,91]. Moreover, EDTA combined with minocycline demonstrated high efficacy in reducing *S. epidermidis*, *S. aureus*, and *C. albicans* embedded in the biofilm adhering to catheter surfaces [92]. Chelating agents destabilize the biofilm matrix by sequestering iron, zinc, magnesium, and calcium [93]. Subsequent studies have indicated that chitosan inhibits biofilm formation, reduces biofilm viability, and disrupts the established biofilm in *Staphylococcus* and *Candida* species [94,95]. Furthermore, hyaluronic acid is also involved in several epithelial tissue regeneration mechanisms, as well as in the regulation of inflammation by CD44 and RHAMM signaling pathways [96]. However, low molecular weight degradation products of hyaluronic acid, resulting from the activity of hyaluronidase-1 (Hyal-1) and hyaluronidase-2 (Hyal-2) enzymes, may present pro-inflammatory properties [96,97]. As shown in Table 2 and Figures S15 and S16, stronger activities towards metal chelating (IC_50_ in the range of 2.97 ± 0.03–3.27 ± 0.03 mg mL^−1^) and hyaluronic acid degradation inhibition (IC_50_ in the range of 0.14 ± 0.00–0.21 ± 0.00 mg mL^−1^) were observed for the binary systems as compared to the pure *S. baicalensis radix* lyophilized extract (IC_50_ = 8.54 ± 0.13 mg mL^−1^ and IC_50_ = 2.00 ± 0.06 mg mL^−1^, respectively). In contrast, chitosan at the concentration from 1 mg mL^−1^ to 10 mg mL^−1^ did not show chelating activity. Due to the formation of high-viscosity chitosan solutions at low pH (4.5), it was impossible to analyze higher chitosan concentrations with a complete mixing of the sample components. *S. baicalensis radix* lyophilized extract in the presence of chitosan showed an increased chelating activity, most likely due to the formation of intermolecular interactions between the extract and chitosan (ATR-FTIR analysis confirmed the formation of hydrogen bonds, Section 3.2.1). However, Shanmugan et al. reported the iron-chelating ability of chitosan from the shells of bivalve mollusk *Donax scortum* in the range of 9.38%–71.06% for the concentrations between 1–10 mg mL^−1^ [98]. Nevertheless, the viscosity of the chitosan did not significantly affect anti-hyaluronidase activities. In our previous studies, we indicated the less active binary mixtures based on chitosan with a higher viscosity [64]. The obtained results may stem from the greater pH-solubility of chitosan in the acetic buffer solution at pH 4.5, which was used to prepare the binary mixtures solutions [99]. Furthermore, electrostatic interactions between the amino group (NH_2_) of the chitosan and the carboxyl group (COOH) of the hyaluronic acid constitute the crucial factor of the high anti-hyaluronidase property of the chitosan molecule [100].

#### 3.4.2. Effects on the Vaginal Microflora

In the presented study, the antimicrobial activity of the binary systems against probiotic and pathogenic microorganisms colonizing the vagina was investigated. The antimicrobial activity of binary systems was evaluated according to their inhibition zone diameter against four species of bacteria (*G. vaginalis*, *S. agalactiae*, *S. aureus*, and *E. coli*) and three species of yeast-like fungi (*C. albicans*, *C. parapsilosis*, and *C. krusei*). The effects of the binary systems on microorganisms from the vaginal fluid were compared with the appropriate amount of pure *S. baicalensis radix* lyophilized extract included in the binary system (Table 3). The results revealed the highest antimicrobial activity for the binary systems in the weight ratio of 2:1, whereas the greatest increase in activity following the combination of *S. baicalensis radix* extract with chitosan was observed against yeast-like fungi, with *C. parapsilosis* as the most sensitive strain. The previous report showed that baicalein reduced the growth and cell viability of *C. albicans* and non-Candida species, such as *C. glabrata*, *C. parapsilosis*, *C. krusei,* and *C*. *guilliermondii* [101,102]. Dai et al. found that the baicalein induced apoptosis was associated with a breakdown of mitochondrial function in *C. albicans* cells [103]. Moreover, baicalein decreased *C. albicans* biofilm formation by down-regulating CSH1 mRNA expression [104], whereas the combination of baicalein with amphotericin B or fluconazole, respectively, either increased the hydrogen-peroxide-induced apoptosis in *C. albicans* cells or inhibited efflux pumps of fluconazole-resistant *C. albicans* [105,106]. Furthermore, baicalin increased apoptosis-like programmed cell death in *C. albicans* by activating the succinate dehydrogenase and Ca^2+^–Mg^2+^ ATPase and damaging the ultrastructure of the pathogenic fungi [107]. Interestingly, baicalin and baicalein also demonstrate broad-spectrum activity against Gram-positive and Gram-negative bacteria, inhibiting bacterial growth by means of destroying bacterial nucleic acid and altering the energy metabolism of the bacterial cell [108]. In fact, baicalein may prevent the formation of bacterial biofilms and contribute to the breakdown of biofilms, consequently reducing enterotoxin A and α-hemolysin produced by staphylococci, thus inhibiting the growth of *S. aureus* [109]. Although the presence of chitosan in most cases caused a slight reduction in the activity against pathogenic bacteria of the binary systems, the action of the binary systems in a weight ratio of 2:1 was comparable to that of metronidazole and clindamycin. The effects of the binary systems on the vaginal probiotic *Lactobacillus* species were also investigated, and it was established that they effectively protect the vagina against colonization of the pathogens by producing antimicrobial agents, predominantly bacteriocins and hydrogen peroxide [110]. Chitosan-based binary systems presented a comparable or weaker activity against *L. jensenii* and *L. plantarum* than standard clindamycin, which may be helpful in the development of new strategies aiming to reduce the recurrence of infections. Moreover, the effect of chitosan against the probiotic and pathogenic microorganisms was also determined. Several reports have investigated and described the mechanisms of action of chitosan on bacteria and fungi [111,112,113,114,115,116,117,118,119,120]. According to the research, the antimicrobial properties of chitosan are strongly correlated to its structure, particularly to the reactive hydroxyl groups at the C3 and C6 positions, physicochemical properties, and the environmental conditions. The mode of the antimicrobial action of chitosan can be classified in terms of the extracellular effects, intracellular effects, or both depending on the target of antimicrobial activity. Due to its high molecular weight (MW), chitosan is generally unable to penetrate the cell wall and cell membrane; therefore, its potential antimicrobial activity involves acting as a chelator for essential metals, preventing the absorption of nutrients from external sources, as well as altering cell permeability. In contrast, low MW chitosan presents extracellular and intracellular antimicrobial activity, thus, affecting RNA, protein synthesis, and mitochondrial function. Moreover, the mode of the antimicrobial action of chitosan is highly dependent on the type of the target microorganism. Gram-positive and Gram-negative bacteria differ in the cell wall structure, where Gram-positive bacteria are characterized with coarser peptidoglycans, and Gram-negative bacteria are enriched in lipopolysaccharide (LPS). In fact, differences in the bacterial cell surface structure also contribute to chitosan sensitivity. Gram-negative bacteria show a more negative charge than Gram-positive bacteria, since LPS is often attached to the phosphorylated groups [121]. In turn, more negatively charged cell surfaces allow for the binding of cationic chitosan to phospholipids when the pH of the environment is below 6.5 [122]. Additionally, research reports suggest that Gram-negative bacteria may be more sensitive to chitosan than Gram-positive bacteria, although according to other studies, it is Gram-positive bacteria that are more sensitive to it [116]. Teichoic acids in Gram-positive bacteria are also negatively charged due to the phosphate groups in their structure [123]. However, deletion of the teichoic acid biosynthetic pathway in *S. aureus* resulted in increased resistance to chitosan [124], which indicates that the mode of action of chitosan is more complex than simple electrostatic interactions. Moreover, unlike Gram-negative bacteria, Gram-positive bacteria have a thick cell wall that may prevent chitosan from binding directly to the cell membrane. Nonetheless, some chitosan oligomers (<5 kDa) penetrate the cell wall and affect DNA/RNA or protein synthesis [59]. Interestingly, reports have shown that chitosan (≤50 kDa) possesses the ability to penetrate the cell wall and inhibit DNA transcription [125]. Thus, although the molecular weight of chitosan plays an important role in targeting, the structure of chitosan also determines its extracellular and/or intracellular antimicrobial activity. Chitosan has also been shown to possess fungicidal activity against several fungal pathogens in plants and humans [119], and its antifungal properties are mainly associated with the interaction of chitosan with the cell wall or cell membrane. Nevertheless, the minimum inhibitory concentrations (MIC) of chitosan against fungi are strongly related to the MW and the degree of the deacetylation (DDA) of chitosan, the pH of the solvent, and the species of fungi causing infection [126]. Moreover, the content of unsaturated fatty acids on the cell membrane may be positively correlated with the sensitivity to chitosan [127], since a higher content of unsaturated fatty acids shows greater membrane fluidity, resulting in a more negative charge on the cell membrane [128]. For instance, the sensitivity of *Neurospora crassa* strains to chitosan is related to the content of unsaturated fatty acids on the cell membranes [127]. These data may at least partially account for the reason why *C. albicans*, *C. tropicalis*, and other *Candida* species show significant differences in susceptibility to the same chitosan [129]. Indeed, *C. tropicalis* showed an over 1000-fold increase in exposure to some chitosans compared to *C. albicans* [120]. Similarly, besides its extracellular antifungal activity, low MW chitosan is able to penetrate the cell wall and the cell surface, leading to the inhibition of DNA/ RNA and protein synthesis [130]. In the presented study, chitosan demonstrated low antibacterial efficacy or was inactive against the studied pathogens at the concentrations of 400–800 µg mL^−1^ with the inhibition zone diameter in the range of 0.0 ± 0.0 to 5.0 ± 0.0 mm (Table S9). Furthermore, the antifungal activity of chitosan with an increase in the dose was also observed. The strongest action against the *Candida* species was found for chitosan 80:1000 at the concentration of 800 µg mL^−1^ with the inhibition zone diameter in the range of 3.0 ± 0.0 to 7.0 ± 0.0 mm, whereas chitosan at the concentration of 400 µg mL^−1^ was inactive. These results indicate that a combination of *S. baicalensis radix* lyophilized extract and chitosan significantly increased the antimicrobial activity of the binary systems against *Candida* species, particularly in the weight ratio SBE/CS of 2:1. Several studies have also observed the synergistic antimicrobial activity of chitosan with other plant materials or natural compounds [131,132,133]. Moreover, the mucoadhesive properties of chitosan may allow for the maintenance of the antimicrobial effect of the binary systems in the vagina environment for a longer period of time.

## 4. Conclusions

A chitosan-based system with *S. baicalensis radix* lyophilized extract is a promising alternative for developing new strategies in the treatment of vaginal infections, particularly vulvovaginal candidiasis. Vaginal administration allows us to bypass the first-pass metabolism, drug interactions, and adverse effects. Drugs that have been clinically used for local treatment of vaginal infections show only antimicrobial or antifungal properties. Therefore, a combination of a *S. baicalensis radix* lyophilized extract with chitosan could improve topical vaginal therapy by decreasing the overgrowth of the common pathogens associated with vaginal infections, as well as reducing inflammation and vaginal dryness. Due to strong iron-chelating properties, it may also be effective in the treatment of infection caused by microbial biofilm formed in vaginal epithelial cells. Moreover, the presence of chitosan in the binary systems may increase the retention time of the drug in the vaginal environment. The binary system containing *S. baicalensis radix* lyophilized extract with chitosan 80:1000 in weight ratio 2:1 was found to be most valuable, with the appropriately controlled dissolution of active compounds and a significant biological activity towards inhibiting the hyaluronic acid degradation, metal chelating activity, and providing beneficial effects on the vaginal microbiome microorganisms..

## Figures and Tables

**Table 1:** Intermediate stability studies of *S. baicalensis radix* lyophilized extract.

Compound	Residual Flavones Content (%)					
	1 Month	2 Months	3 Months	4 Months	5 Months	6 Months
baicalin	93.03 ± 0.58 ^a^	91.23 ± 0.25 ^a^	91.25 ± 1.49 ^a^	90.07 ± 7.19 ^a^	90.76 ± 1.27 ^a^	90.95 ± 0.03 ^a^
baicalein	95.59 ± 0.24 ^a^	93.94 ± 0.55 ^b^	93.11 ± 0.55 ^b^	93.68 ± 0.96 ^b^	92.55 ± 0.07 ^b^	92.88 ± 1.02 ^b^
wogonin	92.34 ± 2.82 ^a^	92.26 ± 0.60 ^a^	92.60 ± 3.59 ^a^	92.78 ± 1.44 ^a^	91.49 ± 1.04 ^a^	91.76 ± 0.21 ^a^

The values are presented as the mean ± SD (*n* = 3). Mean values with the same letter are not significantly different at *p* < 0.05 using Duncan’s multiple range test. “a” of the alphabet stands for the highest values, “b” stands for the statistically significant decreasing values.

**Table 2:** Biological activity towards ferrous ion-chelating and hyaluronidase inhibition of *S. baicalensis radix* lyophilized extract, chitosans, and the binary systems.

Extract or Chitosan System	IC_50_ (mg mL^−1^)	
	Metal Chelating Activity	Hyaluronidase Inhibition
SBE	8.54 ± 0.13 ^d^	2.00 ± 0.06 ^d^
SBE/Cs 80:500 2:1	3.27 ± 0.03 ^c^	0.19 ± 0.00 ^c^
SBE/Cs 80:1000 2:1	3.11 ± 0.05 ^b^	0.21 ± 0.00 ^c^
SBE/Cs 80:500 1:1	3.01 ± 0.12 ^a^	0.14 ± 0.00 ^b^
SBE/Cs 80:1000 1:1	3.06 ± 0.17 ^a,b^	0.15 ± 0.00 ^b^
SBE/Cs 80:500 1:2	2.97 ± 0.03 ^a^	0.15 ± 0.00 ^b^
SBE/Cs 80:1000 1:2	3.14 ± 0.03 ^b^	0.14 ± 0.00 ^b^
Cs 80:500	n.o.	0.03 ± 0.00 ^a^
Cs 80:1000	n.o.	0.03 ± 0.00 ^a^

The results are presented as the mean ± SD (n = 6). Mean values with the same letter are not significantly different at *p* < 0.05 using Duncan’s multiple range test. “a” of the alphabet stands for the lowest values (the higher activity), “b–d” stand for the statistically significant increasing values. n.o.—not observed at the concentration of 1–10 mg mL^−1^.

**Table 3:** Antimicrobial activity of the binary systems.

	Inhibition Zone Diameter (mm)										
Microorganism	SBE (800 µg mL^−1^)	SBE/Cs 2:1 80/500	SBE/Cs 2:1 80/1000	SBE (600 µg mL^−1^)	SBE/Cs 1:1 80/500	SBE/Cs 1:1 80/1000	SBE (400 µg mL^−1^)	SBE/Cs 1:2 80/500	SBE/Cs 1:2 80/1000	Metronidazole	Clindamycin
*Gardnerella vaginalis*	27.0 ± 2.0 ^a^	25.0 ± 2.0 ^a^	21.0 ± 2.0 ^b^	20.0 ± 1.0 ^b^	19.0 ± 2.0 ^b^	16.0 ± 2.0 ^c,d^	17.0 ± 2.0 ^c^	14.0 ± 2.0 ^c,d^	13.0 ± 1.0 ^d^	26.0 ± 1.0 ^a^	25.0 ± 2.0 ^a^
*Streptococcus agalactiae*	28.0 ± 2.0 ^a^	25.0 ± 2.0 ^b,c^	24.0 ± 2.0 ^c^	25.0 ± 2.0 ^b,c^	20.0 ± 2.0 ^d^	18.0 ± 2.0 ^d,e^	18.0 ± 2.0 ^d^	15.0 ± 1.0 ^e^	12.0 ± 1.0 ^f^	27.0 ± 1.0 ^a,b^	25.0 ± 2.0 ^b,c^
*Staphylococcus aureus*	28.0 ± 3.0 ^a^	26.0 ± 2.0 ^a,b^	24.0 ± 2.0 ^b^	26.0 ± 3.0 ^a,b^	20.0 ± 2.0 ^c^	18.0 ± 2.0 ^c,d^	20.0 ± 2.0 ^c,d^	15.0 ± 1.0 ^d^	9.0 ± 1.0 ^e^	27.0 ± 1.0 ^a,b^	25.0 ± 2.0 ^a,b^
*Escherichia coli*	23.0 ± 2.0 ^b,c^	26.0 ± 1.0 ^a,b^	25.0 ± 2.0 ^b^	22.0 ± 2.0 ^c,d^	21.0 ± 2.0 ^c,d,e^	19.0 ± 2.0 ^e,f^	19.0 ± 2.0 ^e,f^	16.0 ± 1.0 ^f^	11.0 ± 1.0 ^g^	28.0 ± 2.0 ^a^	19.0 ± 2.0 ^d,e^
*Lactobacillus gasseri*	22.0 ± 2.0 ^a,b,c^	22.0 ± 2.0 ^a,b,c^	22.0 ± 2.0 ^a,b,c^	20.0 ± 2.0 ^b,c,d^	19.0 ± 2.0 ^c,d^	19.0 ± 2.0 ^d^	20.0 ± 2.0 ^b,c,d^	17.0 ± 1.0 ^d,e^	11.0 ± 1.0 ^f^	23.0 ± 1.0 ^a^	15.0 ± 1.0 ^e^
*Lactobacillus jensenii*	20.0 ± 2.0 ^b,c^	21.0 ± 2.0 ^b^	21.0 ± 2.0 ^b^	18.0 ± 2.0 ^c^	19.0 ± 2.0 ^b,c^	19.0 ± 2.0 ^b,c^	12.0 ± 2.0 ^e^	14.0 ± 1.0 ^d^	11.0 ± 1.0 ^e^	20.0 ± 1.0 ^b,c^	25.0 ± 2.0 ^a^
*Lactobacillus plantarum*	19.0 ± 0.0 ^a^	16.0 ± 1.0 ^b,c^	19.0 ± 1.0 ^a^	17.0 ± 0.0 ^b^	15.0 ± 1.0 ^c^	15.0 ± 1.0 ^c^	11.0 ± 0.0 ^d^	15.0 ± 1.0 ^c^	11.0 ± 1.0 ^d^	16.0 ± 1.0 ^b,c^	19.0 ± 2.0 ^a^
*Candida albicans*	15.0 ± 0.0 ^b^	16.0 ± 1.0 ^b^	18.0 ± 2.0 ^a^	13.0 ± 0.0 ^c^	16.0 ± 1.0 ^b^	18.0 ± 2.0 ^a^	11.0 ± 0.0 ^d^	15.0 ± 1.0 ^b^	12.0 ± 1.0 ^c,d^	18.0 ± 1.0 ^a^	15.0 ± 1.0 ^b^
*Candida parapsilosis*	13.0 ± 0.0 ^d,e^	17.0 ± 1.0 ^c^	22.0 ± 1.0 ^b^	11.0 ± 0.0 ^f,g^	17.0 ± 2.0 ^c^	18.0 ± 2.0 ^c^	10.0 ± 0.0 ^g^	14.0 ± 1.0 ^d^	12.0 ± 1.0 ^e,f^	18.0 ± 1.0 ^c^	25.0 ± 2.0 ^a^
*Candida krusei*	12.0 ± 0.0 ^f^	18.0 ± 1.0 ^d^	20.0 ± 1.0 ^c^	12.0 ± 0.0 ^f^	15.0 ± 1.0 ^d^	18.0 ± 1.0 ^d^	10.0 ± 0.0 ^g^	13.0 ± 1.0 ^f^	12.0 ± 1.0 ^f^	27.0 ± 2.0 ^a^	25.0 ± 2.0 ^b^

All the binary systems were tested at a concentration of 1200 µg mL^−1^. Reference substances, such as metronidazole and clindamycin, were tested at a concentration of 1000 µg mL^−1^. The results are presented as the mean ± SD (*n* = 3). Mean values with the same letter are not significantly different at *p* < 0.05 using Duncan’s multiple range test. “a” of the alphabet stands for the highest values, “b–g” stand for the statistically significant decreasing values.

## Data Availability

The data presented in this study are available upon request from the first author.

## Supplementary Materials

The following supporting information can be downloaded at: https://www.mdpi.com/article/10.3390/pharmaceutics14040740/s1, Figure S1: Experimental and calculated (B3LYP/6-31G (d,p)) ATR-FTIR spectra for baicalin; Figure S2: Experimental and calculated (B3LYP/6-31G (d,p)) ATR-FTIR spectra for baicalein; Figure S3: Experimental and calculated (B3LYP/6-31G (d,p)) ATR-FTIR spectra for wogonin; Figure S4: ATR-FTIR spectra of *S. baicalensis radix* extract and flavones; Figure S5: Second derivative infrared spectra (by the Savitzky–Golay polynomial fitting method, 25-point smoothing) of *S. baicalensis radix* extract and flavones; Figure S6: TG/DTG curve of *S. baiacalensis radix* extract at a heating rate of 10 °C min^−1^; Figure S7: TG/DTG curve of chitosan 80:500 at a heating rate of 10 °C min^−1^; Figure S8: TG/DTG curve of chitosan 80:1000 at a heating rate of 10 °C min^−1^; Figure S9: TG/DTG curve of *S. baiacalensis radix* extract and chitosan 80:500 in weight ratio 2:1 at a heating rate of 10 °C min^−1^; Figure S10: TG/DTG curve of *S. baiacalensis radix* extract and chitosan 80:1000 in weight ratio 2:1 at a heating rate of 10 °C min^−1^; Figure S11: TG/DTG curve of the binary system with *S. baiacalensis radix* extract and chitosan 80:500 in weight ratio 1:1 at a heating rate of 10 °C min^−1^; Figure S12: TG/DTG curve of *S. baiacalensis radix* extract and chitosan 80:1000 in weight ratio 1:1 at a heating rate of 10 °C min^−1^; Figure S13: TG/DTG curve of *S. baiacalensis radix* extract and chitosan 80:500 in weight ratio 1:2 at a heating rate of 10 °C min^−1^; Figure S14: TG/DTG curve of *S. baiacalensis radix* extract and chitosan 80:1000 in weight ratio 1:2 at a heating rate of 10 °C min^−1^; Figure S15: Anti-hyaluronidase activity of *S. baicalensis radix* lyophilized extract, chitosans and the binary systems; Figure S16: Metal chelating activity of *S. baicalensis radix* lyophilized extract and the binary systems; Table S1: Selected characteristic bands of baicalin; Table S2: Selected characteristic bands of baicalein; Table S3: Selected characteristic bands of wogonin; Table S4: Location and band assignment of baicalin bands observed on the (i) theoretical (DFT) and experimental (EXP) spectrum, (ii) second derivative infrared spectrum of baicalin and *S. baicalensis radix* lyophilized extract (see Figure S5)); Table S5: Location and band assignment of baicalein bands observed on the (i) theoretical (DFT) and experimental (EXP) spectrum, (ii) second derivative infrared spectrum of baicalein and *S. baicalensis radix* lyophilized extract (see Figure S5); Table S6: Location and band assignment of wogonin bands observed on the (i) theoretical (DFT) and experimental (EXP) spectrum, (ii) second derivative infrared spectrum of wogonin and *S. baicalensis radix* lyophilized extract (see Figure S5); Table S7: Location of *S. baicalensis radix* lyophilized extract and chitosan systems with chitosan 80:500 bands observed on the spectrum of the binary systems (Figure 1a); Table S8. Summary of the inflection points observed on the DTG curve; Table S9: Antimicrobial activity of chitosan.
