# Supplementary material for: The Chitosan-Based System with Scutellariae baicalensis radix Extract for the Local Treatment of Vaginal Infections
Source: Pharmaceutics. 2022 Mar 29;14(4):740. doi: 10.3390/pharmaceutics14040740 (PMC9028937; doi:10.3390/pharmaceutics14040740)
Supplement: Supplementary file 1 [file pharmaceutics-14-00740-s001.zip › pharmaceutics-1633283-supplementary.pdf]

pharmaceutics

MDPI

Supplementary Materials: The Chitosan-based System with *Scutellariae baicalensis radix* Extract for the Local Treatment of Vaginal Infections

Justyna Chanaj-Kaczmarek, Natalia Rosiak, Daria Szymanowska, Marcin Rajewski, Ewa Wender-Ozegowska and Judyta Cielecka-Piontek *

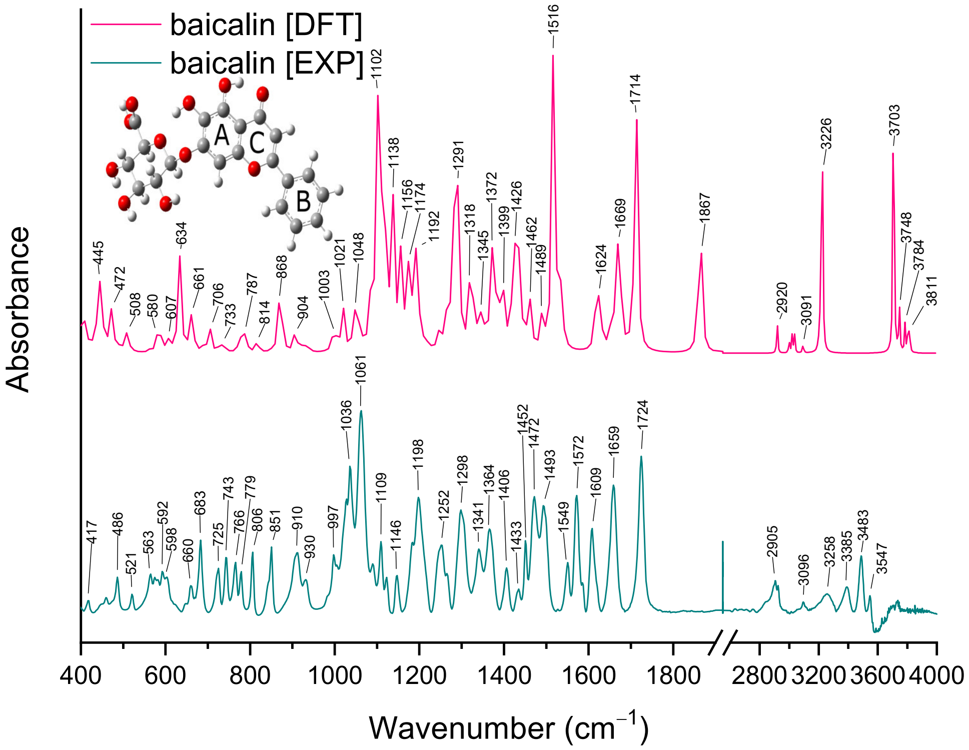

Figure S1. Experimental and calculated (B3LYP/6-31G (d,p)) ATR-FTIR spectra for baicalin.

Calculation and experimental IR absorption spectra of baicalin are displayed in Figure S1. The main characteristic vibrations of the baicalin ATR-FTIR spectra are collected in Table S1. The most intense bands include in the areas 1000–1800 cm^{-1}. Bands derived from 7–*O*–glucuronide predominates in the range 1000–1300 cm^{-1}. For example, the band at 1036 cm^{-1} is related to the stretching vibration of the C–C and the rocking vibration of the C–O–H. At 1061 cm^{-1} is located the band corresponding to the stretching vibration of the C–O–C. Whereas the band at 1109 cm^{-1} is related to the stretching vibration of the C–O. Bands derived from ring A, B or C are located in the range from about 750 to 900 cm^{-1} and 1300–1750 cm−1. For example, the band corresponding to the wagging vibration are located at 766 cm^{-1} (C–H w in ring B), 806 cm^{-1} (C–H w in ring B and C), 851 cm^{-1} (C–H w in ring A), 910 cm^{-1} (C–H w in ring A and C). The bands corresponding to the bending vibration of the C–H bond are located at 1298 cm^{-1} (in ring A), 1406 cm^{-1} (in ring B), 1433 cm^{-1} (in ring B and C) and 1549 cm^{-1} (in ring B). Whereas the bands corresponding to the bending vibration of the C–O–H bond in the A ring are located at 1572 cm^{-1} and 1724 cm−1. But they have additional components corresponding to the bending vibration of the C–H bonds in 7–O–glucuronide (Table S1). The bands related to the stretching vibration are located at 1433 cm^{-1} (C–O–C s in ring C), 1452 cm^{-1} (C–O s in ring A), 1472 cm^{-1} (C=C s in ring A), 1609 cm^{-1} (C=C s in ring A, B, C), 1659 cm^{-1} (C=C s in ring A and C=O s in ring C), 1724 cm^{-1} (C=O s in ring C). Above 2800 cm^{-1} are located the bands associated with the stretching vibration of the C–H and O–H bonds in 7–*O*–glucuronide, ring A, ring B and a hydroxyl group.

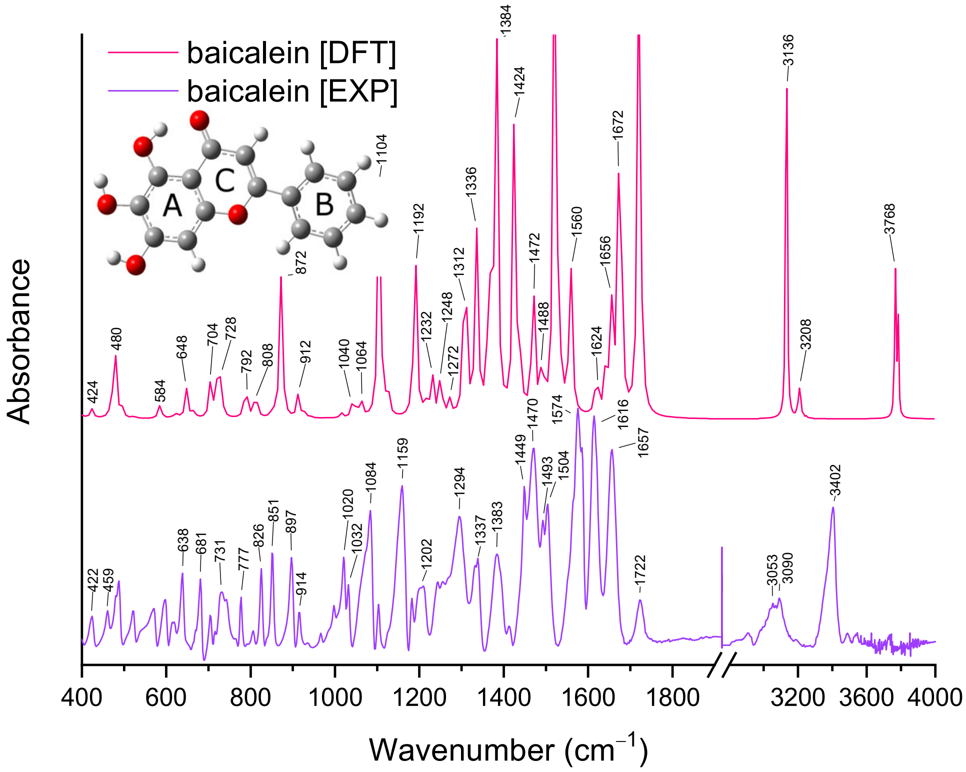

Figure S2. Experimental and calculated (B3LYP/6-31G (d,p)) ATR-FTIR spectra for baicalein.

Calculation and experimental IR absorption spectra of baicalein is displayed in Figure S2. The main characteristic vibrations of the baicalein ATR-FTIR spectra are collected in Table S2. The most intense bands include in the areas 1000–1650 cm^{-1} and they are mainly responsible for the stretching and bending vibration. For example, the band corresponding to the stretching vibration are located at 1020 cm^{-1} (C–O-C in ring C), 1032 cm^{-1} (C-C-C in ring B), 1084 cm^{-1} (C=C in ring C), 1255 cm^{-1} (C-C between C and B ring), 1337 cm^{-1} (C=C in ring C), 1504 cm^{-1} (C–O and C=C in ring A), 1616 cm^{-1} (C=C in ring A and B) and 1657 cm^{-1} (C=C in ring A and C=O in ring C). The bands related to the bending vibration are located at 1020 cm^{-1} (C–H in ring C), 1084 cm^{-1} (C-O–H in ring A and C–H in ring B), 1159 cm^{-1} (C–H in ring B), 1202 cm^{-1} (O–H in ring A, C–H in ring B and C), 1243 cm^{-1} (O–H in ring A), 1255 cm^{-1} (C–H in ring B and C), 1294 cm^{-1} (O–H in ring A), 1337 cm^{-1} (O–H in ring A and C–H in ring B), 1383 cm^{-1} (C-O–H and C–H in ring A), 1449 cm^{-1} (O–H in ring A), 1470 cm^{-1} (O–H in ring A), 1493 cm^{-1} C–H in ring B), 1574 cm^{-1} (C–O–H in ring A) and 1616 cm^{-1} (O–H in ring A). Whereas in the range 400-900 cm^{-1} are located less intense bands corresponding to the wagging vibration (459 cm^{-1} – O–H in ring A, 731 cm^{-1} – C–H in ring B, 826 cm^{-1} – C–H in ring B, 851 cm^{-1} – C–H in ring A and 897 cm^{-1} – O–H in ring A and C–H in ring C) and deformation all molecule (422 cm^{-1}, 638 cm^{-1}, 681 cm^{-1} and 914 cm^{-1}). Above 3000 cm^{-1} are located the bands associated with the stretching vibration of the C–H bond in ring B (3090 cm^{-1}) and O–H bond in ring A (3053 cm^{-1} and 3402 cm^{-1}).

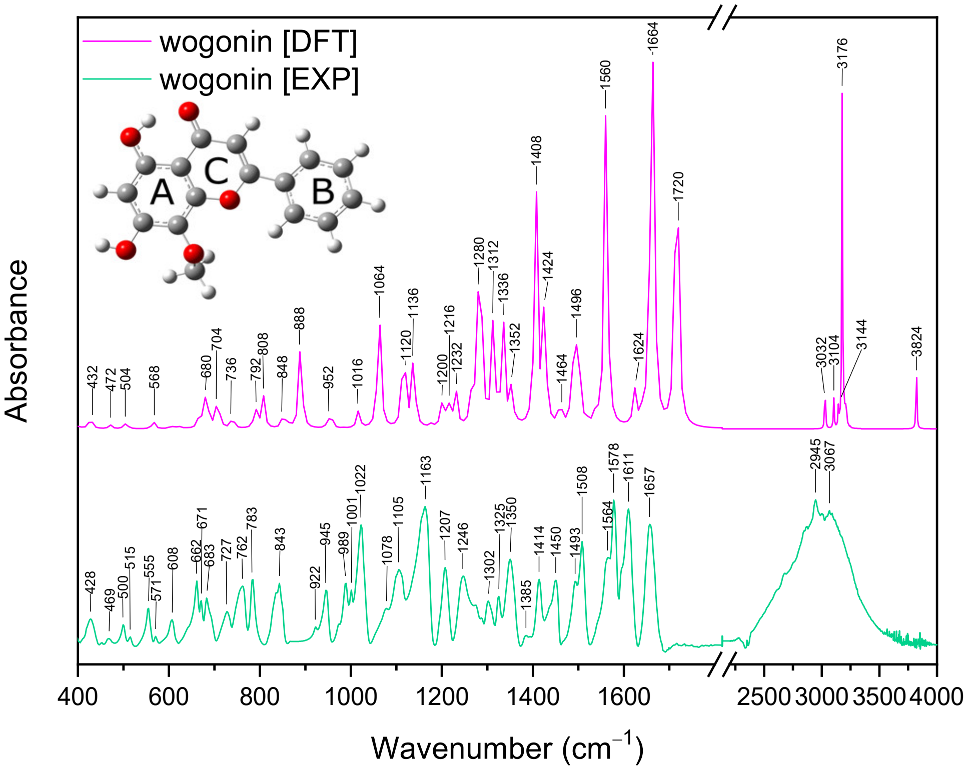

Figure S3. Experimental and calculated (B3LYP/6-31G (d,p)) ATR-FTIR spectra for wogonin.

Calculation and experimental IR absorption spectra of wogonin is displayed in Figure S3. The main characteristic vibrations of the wogonin ATR-FTIR spectra are collected in Table S3. The most intense bands include in the range about 1000–1700 cm^{-1} and they are mainly responsible for the stretching and bending vibration. The bands corresponding to the stretching vibration of the C–C bond are located at 1385 cm^{-1} (in ring A), 1414 cm^{-1} (in ring A and between ring A and C), 1578 cm^{-1} (in ring A, B and C) and 1611 cm^{-1} (in ring A and B). Next the bands corresponding to the stretching vibration of the C=O bond are located at 1385 cm^{-1}, 1611 cm^{-1} and 1657 cm^{-1} (in ring C). For example, the band corresponding to the bending vibration are located at 1105 cm^{-1} (O–H and C–H b in ring A and C–H in ring B), 1163 cm^{-1} (O–H in ring A and C–H in ring B and C), 1207 cm^{-1} (C–H in ring A), 1246 cm^{-1} (O–H and C–H in ring A), 1271 cm^{-1} (C–H in ring B), 1302 cm^{-1} (O–H in ring A and C–H in ring B and C), 1325 cm^{-1} (C–H in ring B and C), 1350 cm^{-1} (C–H in ring B and C), 1385 cm^{-1} (O–H in ring A), 1508 cm^{-1} (C–O–H b + C–H in ring A). Whereas in the range 400-950 cm^{-1} are located less intense bands corresponding to the wagging vibration (500 cm^{-1}, 671 cm^{-1}, 727 cm^{-1}, 762 cm^{-1}, 783 cm^{-1}, 843 cm^{-1} –Table S3), deformation ring (608 cm^{-1} – ring B and C) or all molecule (555 cm^{-1} and 662 cm^{-1}) and breathing ring (428 cm^{-1} – ring A and B, 945 cm^{-1} – ring B). Above 300 cm^{-1} are located the bands associated with the stretching vibration of the C–H bond in CH_3 at ring A (2869 cm^{-1}, 2945 cm^{-1}, 3007 cm^{-1}) and O–H bond at ring A (3067 cm^{-1}).

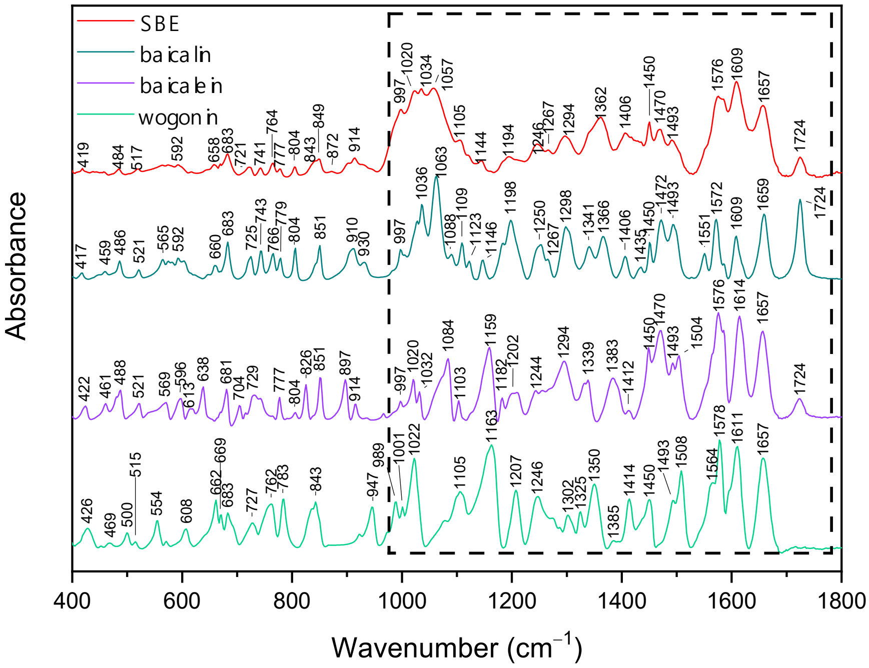

Figure S4. ATR-FTIR spectra of *S. baicalensis radix* extract and flavones.

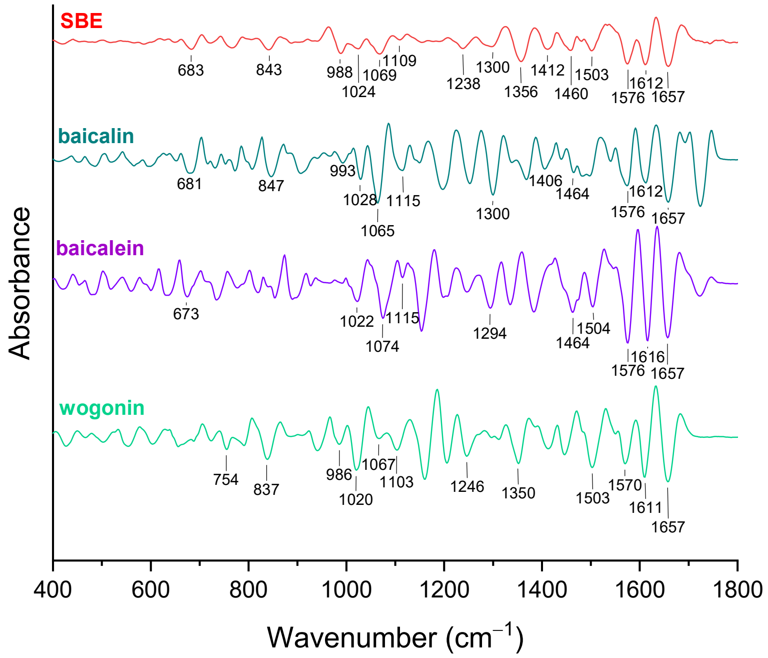

Figure S5. Second derivative infrared spectra (by the Savitzky–Golay polynomial fitting method, 25-point smoothing) of *S. baicalensis radix* extract and flavones.

Second derivative infrared spectra make it possible to identify the bands originating from baicalin, baicalein and wogonin in the *S. baicalensis radix* lyophilized extract spectrum. A summary of the locations of the bands marked in Figure S5 is presented in Tables S4-6.

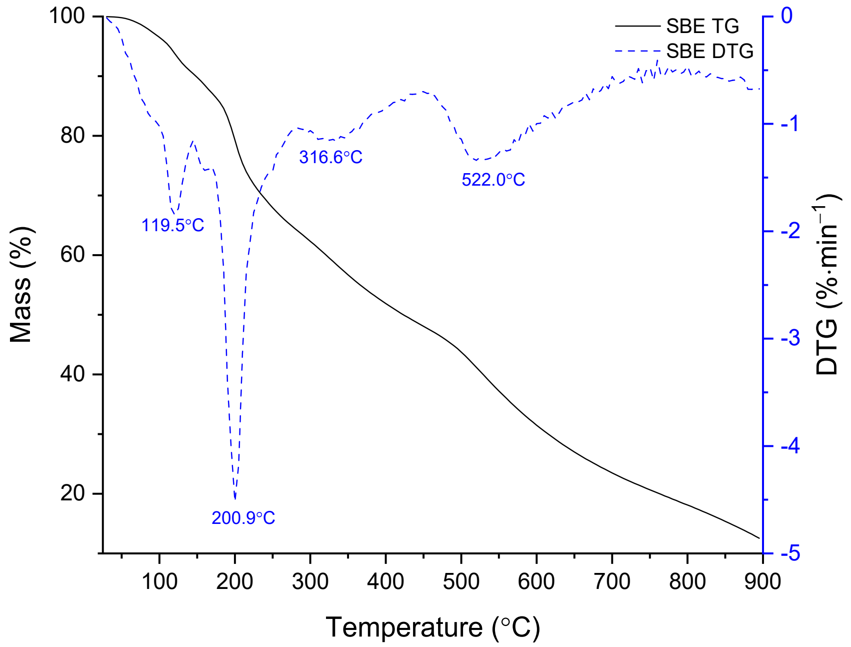

Figure S6. TG/DTG curve of *S. baiacalensis radix* extract at a heating rate of 10 ·min−1.

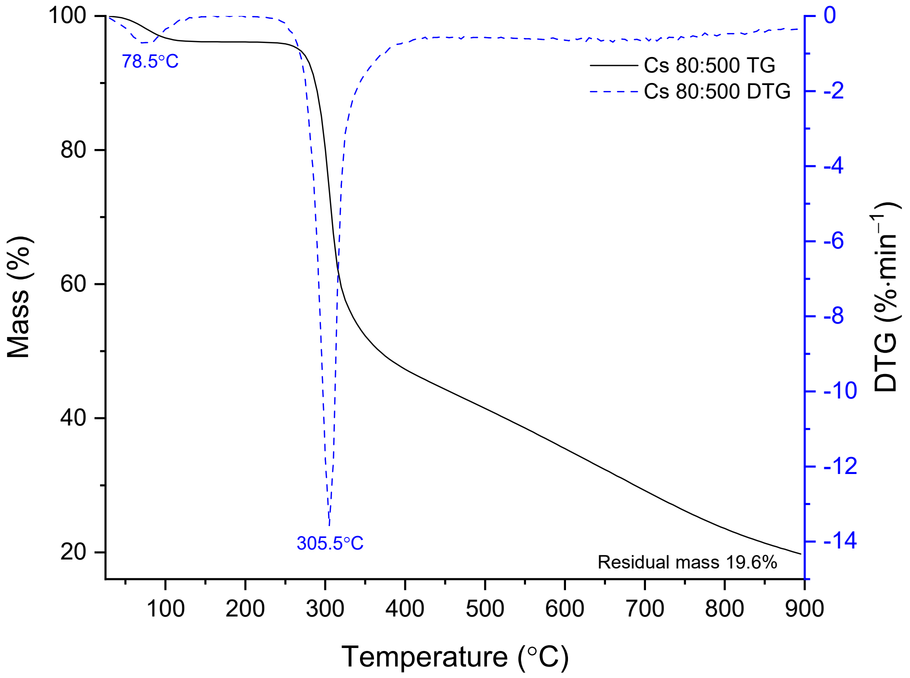

Figure S7. TG/DTG curve of chitosan 80:500 at a heating rate of 10 °C min−1.

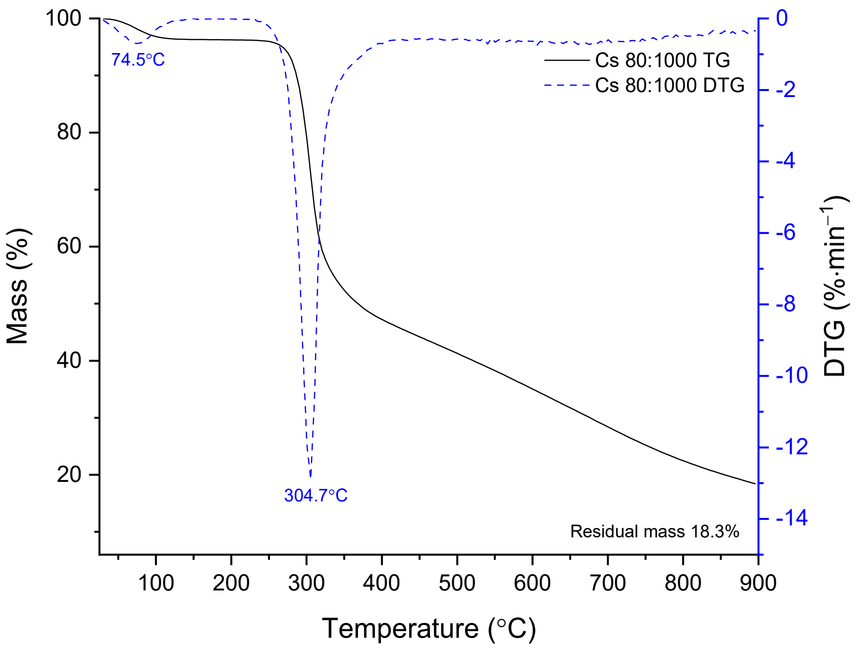

Figure S8. TG/DTG curve of chitosan 80:1000 at a heating rate of 10 °C min^{-1}.

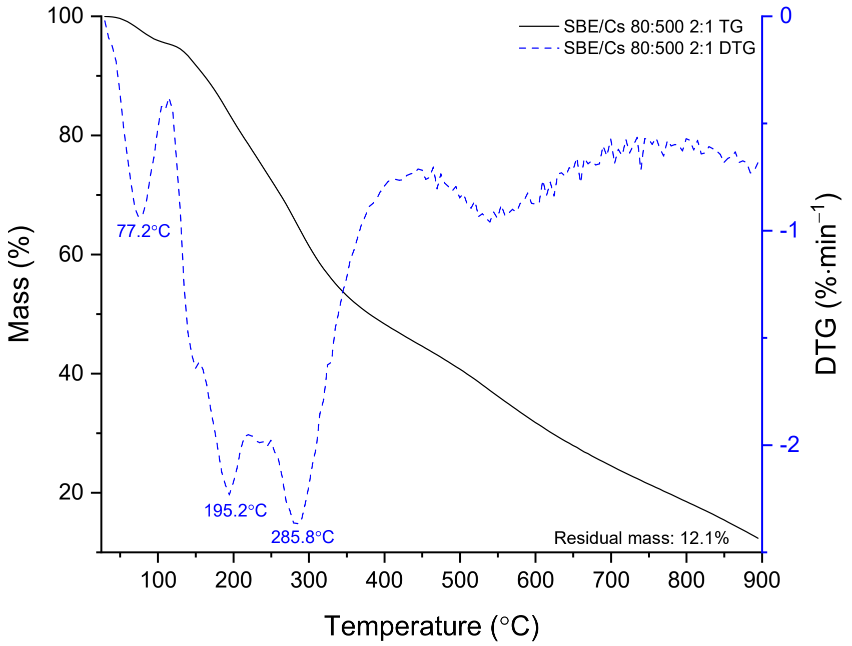

Figure S9. TG/DTG curve of *S. baiacalensis radix* extract and chitosan 80:500 in weight ratio 2:1 at a heating rate of 10 °C min^{-i}.

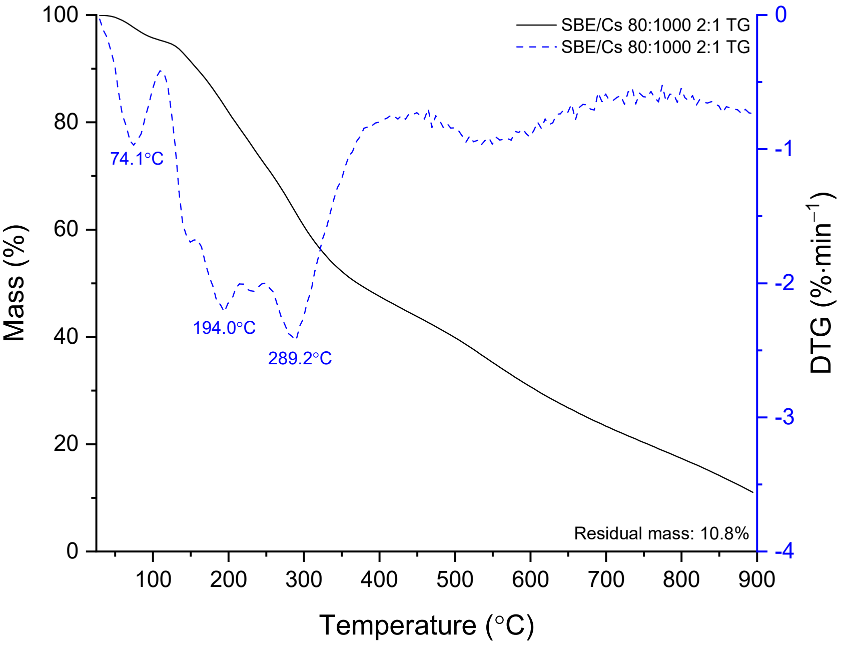

Figure S10. TG/DTG curve of *S. baiacalensis radix* extract and chitosan 80:1000 in weight ratio 2:1at a heating rate of 10 °C min⁻ⁱ.

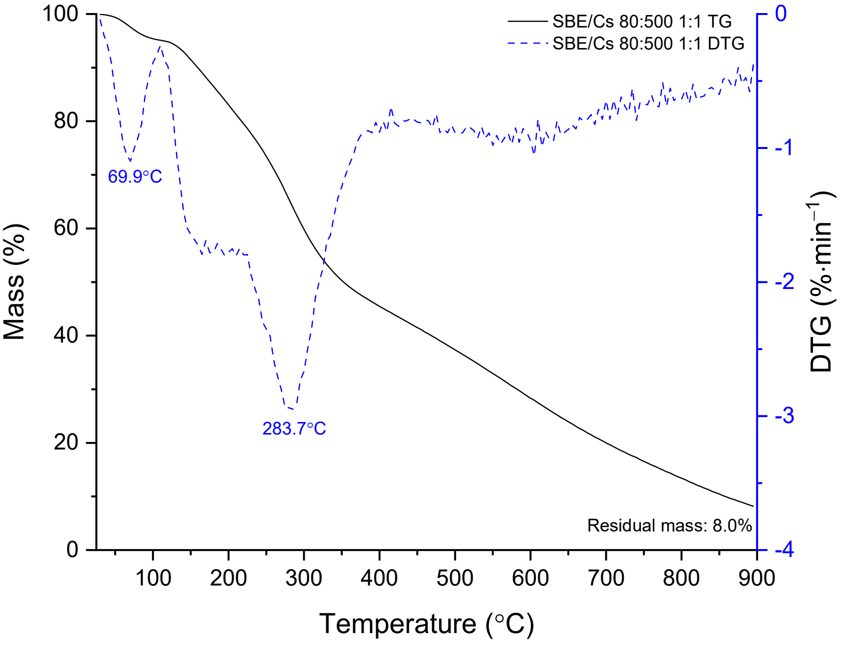

Figure S11. TG/DTG curve of the binary system with *S. baiacalensis radix* extract and chitosan 80:500 in weight ratio 1:1 at a heating rate of 10 °C min^{-1}.

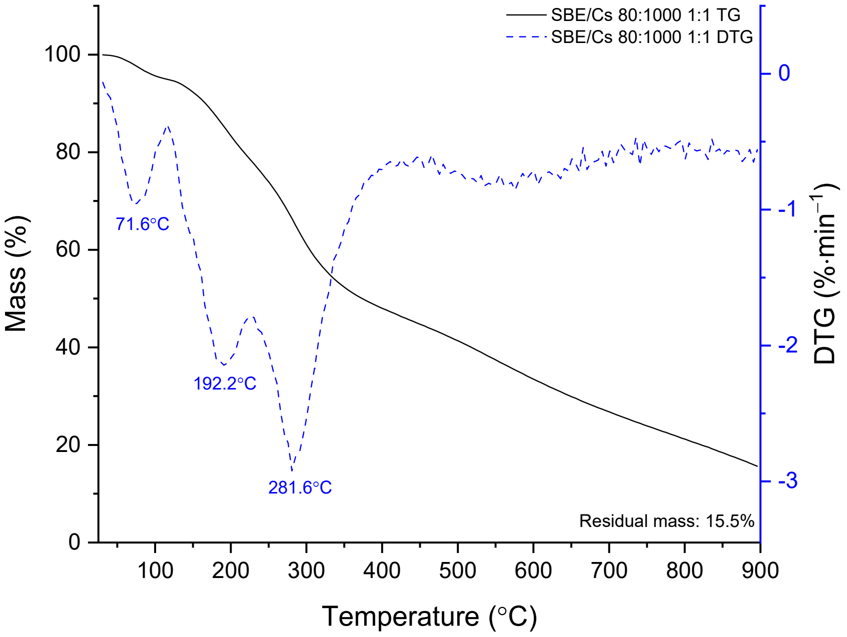

Figure S12. TG/DTG curve of *S. baiacalensis radix* extract and chitosan 80:1000 in weight ratio 1:1at a heating rate of 10 °C min⁻ⁱ.

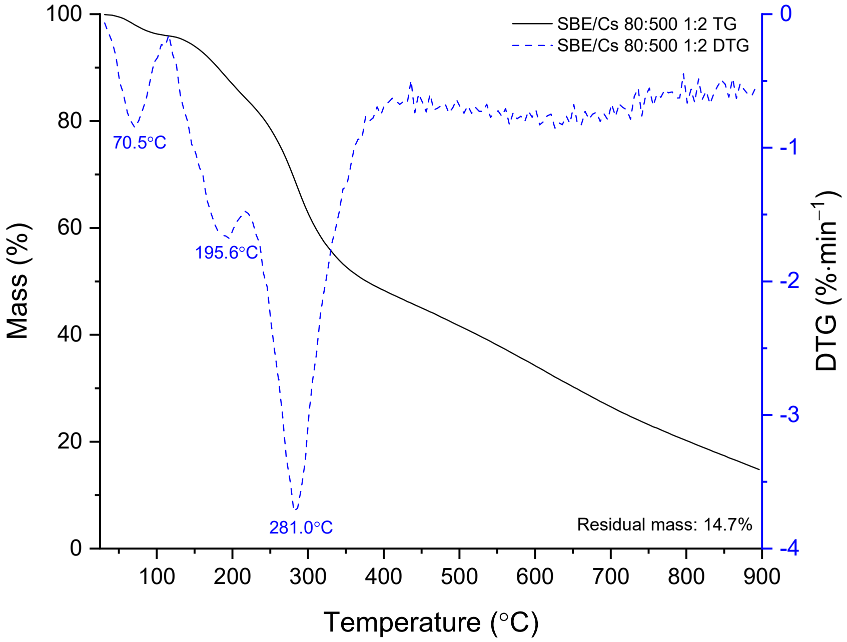

Figure S13. TG/DTG curve of *S. baiacalensis radix* extract and chitosan 80:500 in weight ratio 1:2 at a heating rate of 10 °C min⁻ⁱ.

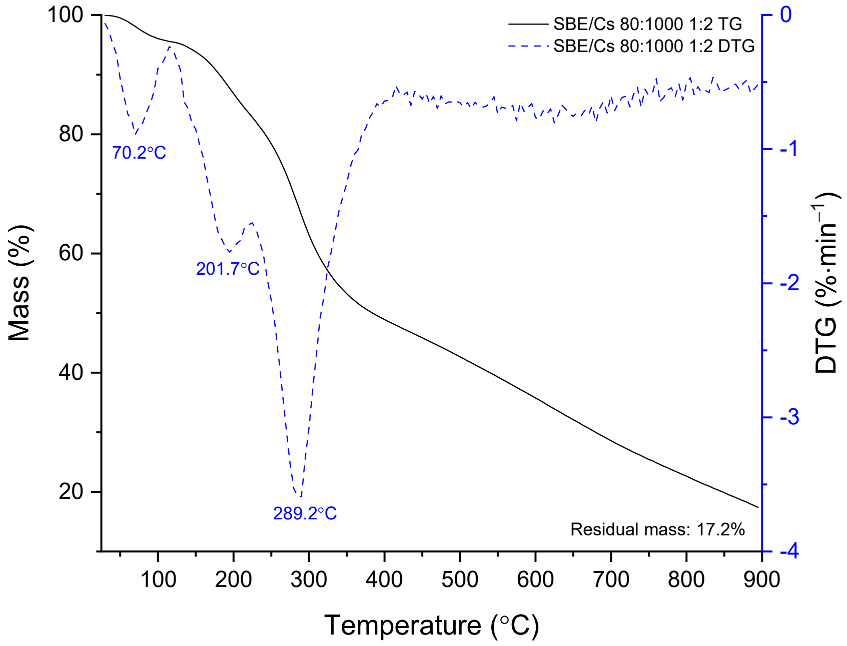

Figure S14. TG/DTG curve of *S. baiacalensis radix* extract and chitosan 80:1000 in weight ratio 1:2 at a heating rate of 10 °C min⁻¹.

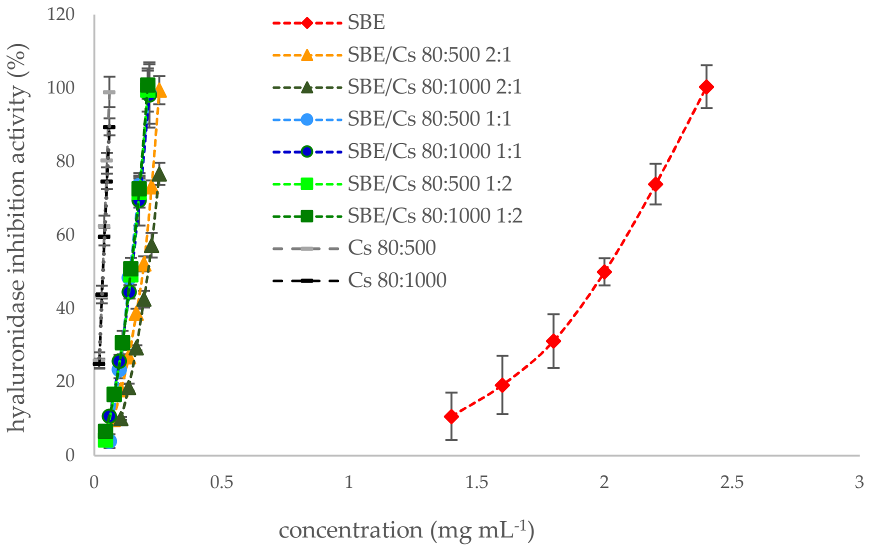

Figure S15. Anti-hyaluronidase activity of *S. baicalensis radix* lyophilized extract, chitosans and the binary systems.

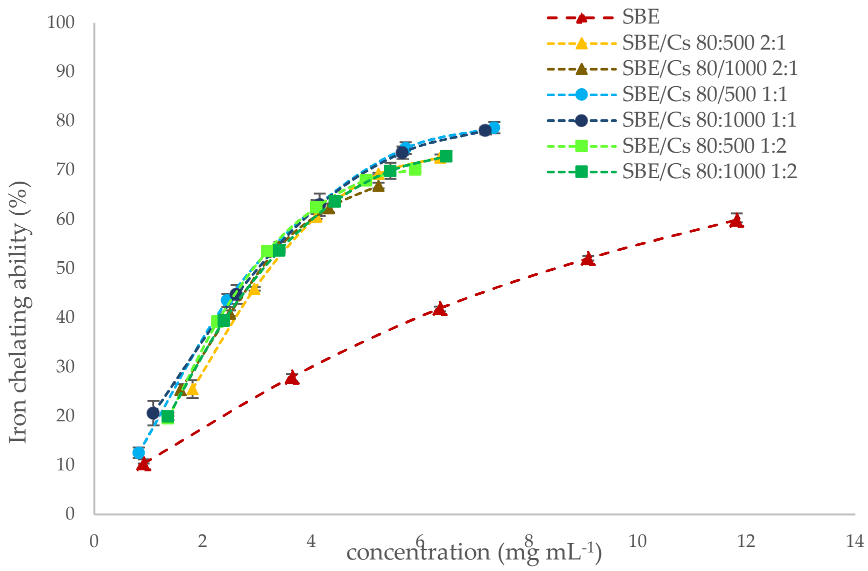

Figure S16. Metal chelating activity of *S. baicalensis radix* lyophilized extract and the binary systems.

Table S1. Selected characteristic bands of baicalin.

DFT	EXP	
411	417	C-C-C t (A)
445	486	O–H w (7–*O*–glucuronide)
472	521	O–H w (7–*O*–glucuronide)
	563	
508	592	O–H w (B) + def. 7–*O*–glucuronide structure
	598	
580	660	def. all molecule
607	683	def. all molecule
634	725	O–H w in COO–H (7–*O*–glucuronide)
661	743	def. all molecule
706	766	C–H w (B)
733	779	C–O–H w + CC–H t (7–*O*–glucuronide)
787	806	C–H w (B, C)
814	851	C–H w (A)
868	910	O–H w (A) + C–H w (C)
904	930	breathing ring (A) + C–C–C s (C)
1003	997	C–H t (A)
1021	1036	C–O–H r + CC s (7–*O*–glucuronide)

1048	1061	C–O-C s (7–*O*–glucuronide)
1102	1109	CO s (7–*O*–glucuronide)
1138	1146	C–O–H b (7–*O*–glucuronide)
1156	1198	C–H b + COO s + CO s + O–H b (7–*O*–glucuronide) + C–H b (A–C)
1174	1252	C–H b (A, C) + C–H b and CO–H b (7–*O*–glucuronide)
1192	1298	C–H b (A) + O–H b (7–*O*–glucuronide)
1291	1341	breathing ring (B) + CCC asym. s (A, C) + O–H b (A)
1318	1364	C–H b + CO b (7–*O*–glucuronide)
1345	1406	C–H b (B) + breathing ring (A)
1372	1433	C–H b (B) + C–O-C s + C–H b (C)
1399	1452	O–H b + C-O s (A) + C–H b (7–*O*–glucuronide)
1426	1472	O–H b + C=C s (A) + C–H b (7–*O*–glucuronide)
1462	1493	C–H b (7–*O*–glucuronide)
1489	1549	C–H b (B)
1516	1572	C–O–H b (A)
1624	1609	C=C s (A, B, C)
1669	1659	C=C s (A) + CO s (C)
1714	1724	C–O–H b (A) + CO s (C)
2920	2905	C–H s (7–*O*–glucuronide)
3001<br>3019<br>3037	3096	C–H s (7–*O*–glucuronide)
3091	3258	C–H s (7–*O*–glucuronide)
3226	3385	C–H s (B)
3703	3483	O–H s (A)
3748	3547	O–H s in COO–H (7–*O*–glucuronide)

s—stretching, b—bending, w—wagging, t—twisting, def.—deformation, A–ring A, B—ring B, C—ring C.

Table S2. Selected characteristic bands of baicalein.

DFT	EXP	
424	422	def. all molecule
480	459	O–H w (A)
584	638	def. all molecule
648	681	def. all molecule

704	731	C–H w (B)
728	777	C-C-C t (C)
792	826	C–H w (B)
808	851	C–H w (A)
872	897	O–H w (A) + C–H w (C)
912	914	def. all molecule
1040	1020	breathing ring (B) + C–O-C s + C–H b (C)
1064	1032	C-C-C s (B)
1104	1084	C-O–H b (A) + C–H b (B) + C=C s (C)
1192	1159	C–H b (B)
1232	1202	O–H b (A) + C–H b (B) + C–H b (C)
1248	1243	O–H b (A)
1272	1255	C–H b (C) + C-C s (C–B) + C–H b (B)
1312	1294	O–H b + breathing ring (A)
1336	1337	O–H b (A) + C–H b (B) + C=C s (C)
1384	1383	C-O–H b +C–H b (A)
1424	1449	O–H b + breathing ring (A)
1472	1470	O–H b (A)
1488	1493	C–H b (B)
1519	1504	C–O s + C=C s (A)
1560	1574	C–O–H b (A)
1656	1616	C=C s (A, B) + O–H b (A)
1672	1657	C=C s (A) + C=O s (C)
1725	1722	C=O s (C) + C–O–H b (A)
3136	3053	O–H s (A)
3208	3090	C–H s (B)
3768	3402	O–H s (A)

s—stretching, b—bending, w—wagging, t—twisting, def.—deformation, A–ring A, B—ring B, C—ring C.

Table S3. Selected characteristic bands of wogonin.

**DFT**	**EXP**	
432	428	breathing ring (A, B)
472	500	C–C–C w + C–H w (B)
504	555	def. all molecule

568	608	def. B and C ring
680	662	def. all molecule
704	671	C–H w (B)
736	683	C–C–C t (C)
792	727	C–H w (B)
808	762	C–H w (A)
848	783	C–H w (B, C)
888	843	O–H w (A)
952	945	breathing ring (B)
1016	1001<br>989	breathing ring (A, B, C) + C–H b (C)
1064	1022	C–O s in methoxy group (A) + C–H b (A, B, C)
1120	1105	O–H b + C–H b + breathing ring (A) + C–H b (B)
1136	1163	C–C–C s + O–H b (A) + C–H b (B, C)
1232	1207	C–H_2 w in CH_3 + C–H b (A)
1280	1246	O–H b + C–H b (A)
1312	1271	C–C–C s (A, C) + C–H b (C)
1336	1302	O–H b (A) + CC s (A, B) + C–H b (B, C)
1352	1325	breathing ring (A) + C–H b (B, C)
1408	1350	breathing ring (A) + C–H b (B)
1424	1385	O–H b + CC s (A) + CO s (C)
1464	1414	C–H_3 w + O–H b + CC s (A) + CC s (A-C)
1496	1450	O–H b + C–H_2 b in in CH_3 (A)
1560	1508	C–O–H b + C–H b (A)
1624	1578	C–C s (A, B, C)
1664	1611	C–C s (A, B) + C=O s (C)
1720	1657	C–O–H b (A) + C=O s (C)
3032	2869	C–H s in CH_3 (A)
3104	2945	C–H s in CH_3 (A)
3144	3007	C–H s in CH_3 (A)
3176	3067	O–H s (A)

s—stretching, b—bending, w—wagging, t—twisting, def.—deformation, A–ring A, B—ring B, C—ring C.

Table S4. Location and band assignment of baicalin bands observed on the (i) theoretical (DFT) and experimental (EXP) spectrum, (ii) second derivative infrared spectrum of baicalin and *S. baicalensis radix* lyophilized extract (see Figure S5).

Bands of baicalin (cm^{-1})				
baicalin		second derivative infrared spectra		band assignment
DFT	EXP	baicalin	SBE	
1669	1659	1657	1657	C=C s (A) + C=O s (C)
1624	1609	1612	1612	C=C s (A, B, C)
1516	1572	1576	1576	C–O–H b (A)
1399 or 1426	1452 or 1472	1464	1460	O–H b + C-O s (A) + C–H b (7–*O*–glucuronide)
1345	1406	1406	1412	C–H b (B) + breathing ring (A)
1192	1298	1300	1300	C–H b (A) + O–H b (7–*O*–glucuronide)
1102	1109	1115	1109	CO s (7–*O*–glucuronide)
1048	1061	1065	1069	C–O-C s (7–*O*–glucuronide)
1003	997	993	988	C–H t (A)
814	851	847	843	C–H w (A)
607	683	681	683	def. all molecule

s—stretching, b—bending, w—wagging, t—twisting, def.—deformation, A–ring A, B—ring B, C—ring C

Table S5. Location and band assignment of baicalein bands observed on the (i) theoretical (DFT) and experimental (EXP) spectrum, (ii) second derivative infrared spectrum of baicalein and *S. baicalensis radix* lyophilized extract (see Figure S5).

Bands of baicalein (cm^{-1})				
baicalein		second derivative infrared spectra		band assignment
DFT	EXP	baicalein	SBE	
1672	1657	1657	1657	C=C s (A) + C=O s (C)
1656	1616	1616	1612	C=C s (A, B) + O–H b (A)
1519	1504	1504	1503	C–O s + C=C s (A)
1472	1470	1464	1460	O–H b (A)
1312	1294	1294	1300	O–H b + breathing ring (A)
1104	1084	1115	1109	C-O–H b (A) + C–H b (B) + C=C s (C)
1192	1159	1074	1069	C–H b (B)
1040	1020	1022	1024	breathing ring (B) + C–O-C s + C–H b (C)
648	681	673	683	def. all molecule

s—stretching, b—bending, w—wagging, t—twisting, def.—deformation, A–ring A, B—ring B, C—ring C

Table S6. Location and band assignment of wogonin bands observed on the (i) theoretical (DFT) and experimental (EXP) spectrum, (ii) second derivative infrared spectrum of wogonin and *S. baicalensis radix* lyophilized extract (see Figure S5).

**Bands of wogonin (cm^{-1})**				
**wogonin**		**second derivative infrared spectra**		**band assignment**
**DFT**	**EXP**	**wogonin**	**SBE**	
808	762	754	766	C–H w (A)
888	843	837	843	O–H w (A)
1016	1001 or 989	986	988	breathing ring (A, B, C) + C–H b (C)
1064	1022	1020	1024	C–O s in methoxy group (A) + C–H b (A, B, C)
1120	1105	1103	1109	O–H b + C–H b + breathing ring (A) + C–H b (B)
1280	1246	1246	1238	O–H b + C–H b (A)
1408	1350	1350	1356	breathing ring (A) + C–H b (B)
1560	1508	1503	1503	C–O–H b + C–H b (A)
1624	1578	1570	1576	C–C s (A, B, C)
1664	1611	1611	1612	C–C s (A, B) + C=O s (C)
1720	1657	1657	1657	C–O–H b (A) + C=O s (C)

s—stretching, b—bending, w—wagging, t—twisting, def.—deformation, A–ring A, B—ring B, C—ring C

Table S7. Location of *S. baicalensis radix* lyophilized extract and chitosan systems with chitosan 80:500 bands observed on the spectrum of the binary systems (Figure 1A).

		SBE/Cs 80:500			
**SBE**	**Cs 80:500**	**2:1**	**1:1**	**1:2**	
683		685↓	685↓	685↓	def. all molecule – baicalin or baicalein
764		↓	↓	↓	C–H b [76]
849		↓	↓	↓	C–H w (A) – baicalin or O–H w (A) – wogonin
914		-	-	-	*
998		-	-	-	C–H t (A) – baicalin or C–H b (C) – wogonin
1023		-	-	-	C–O-C s + C–H b (C) – baicalein or C–O s in methoxy group (A) + C–H b (A, B, C) – wogonin
	1026	1024	1024	1024	C–O s [76]
1058		1056	1063	1063	C–O-C s (7–O–glucuronide) – baicalin or C–H b (B) – baicalein
	1061				C–O s [76]
	1150	1151	1151	1151	C–O–C asymmetric s [76]
1194		↓	-	-	C–C skeleton vibration [76]
1246		↓	↓	↓	O–H b + C–H b (A) – wogonin
1298		1294	-	-	C–H b (A) + O–H b (7–O–glucuronide) – baicalin or O–H b baicalein
	1314	-	1304	1310	C–N s of amide III [76]
1362		1365	-	-	C–H b (B) – wogonin
1406		1406	1406	1406	C–H b (B) – baicalin
1450		1452↓	1452↓	-	O–H b + C-O s (A) + C–H b (7–O–glucuronide) – baicalin or O–H b (A) – baicalein
1470		1470↓	-	-	
1493		-	-	-	C–O s + C=C s (A) – baicalein or C–O–H b + C–H b (A) – wogonin
1576		1574	1560	1558	C–O–H b (A) – baicalin or C–C s (A, B, C) – wogonin
	1587	changes observed			N–H, of the primary amide [76]
1609		1614↓	1614↓	↓	C=C s (A, B, C) – baicalin or C=C s (A, B) + O–H b (A) – baicalein or C–C s (A, B) + C=O s (C) – wogonin
	1652	-	1652	1652	C=O s of amide I [76]
1657		1652	-	-	C=C s (A) + C=O s (C) – baicalin or C=C s (A) + C=O s (C) – baicalein or C–C s (A, B) + C=O s (C) wogonin
1724		-	-	-	*
	2870	2882	2883	2876	C–H symmetric s [76]
2911					*
3329		3358	3358	3358	*
	3358				O–H s [76]

↓ - we observe a decrease in band intensity, * - information about peaks was not found in the literature, - - no bands.

Table S8. Summarry of the inflection points observed on the DTG curve.

	**Number of point**			
**No.**	**1**	**2**	**3**	**4**
SBE	119.5	200.9	316.6	522
Cs 80:500	78.5	305.5	-	-
Cs 80:1000	74.5	304.7	-	-
SBE/Cs 80:500 2:1	77.2	195.2	285.8	~550
SBE/Cs 80:1000 2:1	74.1	194.0	289.2	~550
SBE/Cs 80:500 1:1	69.9	~190	283.7	-
SBE/Cs 80:1000 1:1	71.6	192.2	281.6	-
SBE/Cs 80:500 1:2	70.5	195.6	281.0	-
SBE/Cs 80:1000 1:2	70.2	201.7	289.2	-

Table S9. Antimicrobial activity of chitosan.

Microorganism	Diameter of inhibition zone (mm)					
	400 μg mL^{-1} (SBE/Cs 2:1)		600 μg mL^{-1} (SBE/Cs 1:1)		800 μg mL^{-1} (SBE/Cs 1:2)	
	CS 80:500	Cs 80:1000	Cs 80:500	Cs 80:1000	Cs 80:500	Cs 80:1000
Gardnerella vaginalis	$0.0±0.0^{c}$	$0.0±0.0^{c}$	$2.0±0.0^{b}$	$2.0±0.0^{b}$	$2.0±0.0^{b}$	$5.0±0.0^{a}$
Streptococcus agalactiae	$0.0±0.0^{c}$	$0.0±0.0^{c}$	$2.0±0.0^{b}$	$2.0±0.0^{b}$	$2.0±0.0^{b}$	$4.0±0.0^{a}$
Staphylococcus aureus	$0.0±0.0^{c}$	$0.0±0.0^{c}$	$2.0±0.0^{b}$	$2.0±0.0^{b}$	$2.0±0.0^{b}$	$3.0±0.0^{a}$
Escherichia coli	$0.0±0.0^{c}$	$0.0±0.0^{c}$	$2.0±0.0^{b}$	$2.0±0.0^{b}$	$2.0±0.0^{b}$	$3.0±0.0^{a}$
Lactobacillus gasseri	$0.0±0.0^{b}$	$0.0±0.0^{b}$	$2.0±0.0^{a}$	$2.0±0.0^{a}$	$2.0±0.0^{a}$	$2.0±0.0^{a}$
Lactobacillus jensenii	$0.0±0.0^{d}$	$0.0±0.0^{d}$	$1.0±0.0^{c}$	$2.0±0.0^{b}$	$2.0±0.0^{b}$	$5.0±0.0^{a}$
Lactobacillus plantarum	$0.0±0.0^{d}$	$0.0±0.0^{d}$	$1.0±0.0^{c}$	$2.0±0.0^{b}$	$1.0±0.0^{c}$	$3.0±0.0^{a}$
Candida albicans	$0.0±0.0^{d}$	$0.0±0.0^{d}$	$1.0±0.0^{c}$	$2.0±0.0^{b}$	$1.0±0.0^{b}$	$7.0±0.0^{a}$
Candida parapsilosis	$0.0±0.0^{d}$	$0.0±0.0^{d}$	$1.0±0.0^{c}$	$2.0±0.0^{b}$	$1.0±0.0^{c}$	$3.0±0.0^{a}$
Candida krusei	$0.0±0.0^{d}$	$0.0±0.0^{d}$	$1.0±0.0^{c}$	$2.0±0.0^{b}$	$1.0±0.0^{c}$	$3.0±0.0^{a}$

The results are presented as the mean ± SD (n=3). Mean values with the same letter are not significantly different at $p < 0.05$ using Duncan's multiple range test. "a" of the alphabet stands for the highest values, "b–d" stand for statistically significant decreasing values.
